# Persistent organochlorine pesticides and polychlorinated biphenyls in air of the North Sea region and air-sea exchange

**DOI:** 10.1007/s11356-016-7530-3

**Published:** 2016-09-12

**Authors:** Carolin Mai, Norbert Theobald, Heinrich Hühnerfuss, Gerhard Lammel

**Affiliations:** 1 Federal Maritime and Hydrographic Agency (BSH), Bernhard-Nocht-Str. 78, 20359 Hamburg, Germany; 2 Department of Chemistry, University of Hamburg, Martin-Luther-King-Platz 6, 20146 Hamburg, Germany; 3 Multiphase Chemistry Department, Max Planck Institute for Chemistry, Hahn-Meitner-Weg 1, 55128 Mainz, Germany; 4 Research Centre for Toxic Compounds in the Environment, Masaryk University, Kamenice 5, 62500 Brno, Czech Republic

**Keywords:** Air-sea exchange, North Sea, Organochlorine pesticides, Polychlorinated biphenyls

## Abstract

Organochlorine pesticides (OCPs) and polychlorinated biphenyls (PCBs) were studied to determine occurrence, levels and spatial distribution in the marine atmosphere and surface seawater during cruises in the German Bight and the wider North Sea in spring and summer 2009–2010. In general, the concentrations found in air are similar to, or below, the levels at coastal or near-coastal sites in Europe. Hexachlorobenzene and α-hexachlorocyclohexane (α-HCH) were close to phase equilibrium, whereas net atmospheric deposition was observed for γ-HCH. The results suggest that declining trends of HCH in seawater have been continuing for γ-HCH but have somewhat levelled off for α-HCH. Dieldrin displayed a close to phase equilibrium in nearly all the sampling sites, except in the central southwestern part of the North Sea. Here atmospheric deposition dominates the air-sea exchange. This region, close to the English coast, showed remarkably increased surface seawater concentrations. This observation depended neither on riverine input nor on the elevated abundances of dieldrin in the air masses of central England. A net depositional flux of *p*,*p′-*DDE into the North Sea was indicated by both its abundance in the marine atmosphere and the changes in metabolite pattern observed in the surface water from the coast towards the open sea. The long-term trends show that the atmospheric concentrations of DDT and its metabolites are not declining. Riverine input is a major source of PCBs in the German Bight and the wider North Sea. Atmospheric deposition of the lower molecular weight PCBs (PCB28 and PCB52) was indicated as a major source for surface seawater pollution.

**Electronic supplementary material:**

The online version of this article (doi:10.1007/s11356-016-7530-3) contains supplementary material, which is available to authorized users.

## Introduction

Anthropogenic chemicals, which are persistent and thus resist degradation in the environment, pose a long-term hazard for ecosystems on a large spatial scale (i.e. far beyond the area of initial release). For semi-volatile substances (i.e. those with a vapour pressure over the subcooled liquid in the range 10^−6^–10^−2^ Pa at 20 °C), the long-range atmospheric transport is enhanced by the multi-hopping potential (subsequent cycles of atmospheric transport, deposition and re-volatilisation from land or sea surfaces; grasshopper effect; Gouin et al. 2004; Semeena and Lammel 2005) and their spatial range is often even global (Wania and Mackay 1993; Leip and Lammel 2004). For this reason, a number of persistent organic pollutants (POPs) are restricted under internationally binding regulations, i.e. the global Stockholm Convention on Persistent Organic Pollutants (UNEP 2010) and the POPs protocol to the regional Convention on Long-range Transboundary Air Pollution (UNECE 1998).

The exposure of the marine environment to POPs, both of the global ocean (e.g. Atlas and Giam 1981; Iwata et al. 1993) and of the regional seas (Lipiatou and Saliot 1991; Axelman et al. 1995), has been reported for more than two decades. However, to date, there is still no long-term monitoring established (Lohmann and Muir 2010). Instead, the assessment has to rely on ship cruises and measurements at coastal sites. The North Sea had been found to receive large amounts of organochlorine pesticides (OCPs) and polychlorinated biphenyls (PCBs), but in recent decades, decaying trends in seawater (due to decreasing primary and secondary emissions) have been observed (Brockmann et al. 1994; BSH 2009, 2013). Besides riverine input, dry and wet atmospheric depositions are also input pathways for the pollution of seawater by POPs (Ilyina et al. 2008; O’Driscoll et al. 2013). POP cycling in shelf seas includes transport by winds and sea currents, atmospheric deposition and re-volatilisation from surface seawater, sinking and re-suspension with suspended particulate matter (SPM), phase partitioning in air (aerosol particles) and seawater (SPM), alongside other processes. This is connected to the carbon cycle (Bidleman and McConnell 1995; Lohmann et al. 2007; O’Driscoll et al. 2013).

In the North Sea region, POPs have been measured in rainwater (Baart et al. 1995; Hühnerfuss et al. 1997; Bethan et al. 2001) and surface seawater (Faller et al. 1991; BSH 2009, 2013) and also bioaccumulating along the marine food chain (Falandysz et al. 1994; Bruhn et al. 1999; Dittmann et al. 2012). So far, however, unlike in other European seas, few POP measurements have been made in the atmosphere above the North Sea. In the framework of several annual monitoring cruises performed by the Federal Maritime and Hydrographic Agency of Germany (BSH), the riverine input of OCPs and PCBs and their spatial distribution in surface seawater has been well investigated in the last decades (BLMP 2005; BSH 2009, 2013). Complementary to the seawater-monitoring programme, this study investigated the spatial and seasonal distribution of OCPs and PCBs in the marine atmosphere in 2009 and 2010 in order to assess their cycling in the North Sea region, to verify long-term trends and to advance knowledge of the contribution of atmospheric deposition to surface seawater contamination.

## Methods

### Air sampling and sample preparation

In short, high-volume air samplers (Digitel DHM-60, Riemer, Hausen, Germany) were operated on the top deck of research vessels. In order to sample particulate and gaseous fractions of organics in air separately, these were equipped with glass fibre filters (GFFs, MN85/90BF of 15 cm diameter, Macherey-Nagel, Düren, Germany) and adsorber cartridges (ORBO 2500, Supelco, Sigma-Aldrich, Taufkirchen, Germany) consisting of a sandwich of polyurethane foam (PUF) and XAD-2. More experimental details are described in Mai (2012) and Theobald et al. (2013).

Seasonal variations of organics in air were investigated by means of PUF disc passive air sampling (PAS; Klánová et al. 2006; Yusà et al. 2009) at a residential land based sampling site (Hamburg-Sülldorf, ≈100 km from the North Sea coast) and at a rural coastal sampling site (Tinnum/Sylt) between October 2009 and December 2010. The PUF discs were simultaneously exchanged in fixed time intervals of 28 days. Sampling rates were controlled by the recoveries of performance reference compounds (PRCs) spiked prior to exposure (Water sampling and sample preparation) and are reported in ng/sample.

PUF plugs, PUF discs and XAD-2 were rinsed with tap water and underwent Soxhlet extraction with acetone, hexane and methanol for 12 h each. The pre-cleaned adsorbent materials were dried in a vacuum desiccator for periods ranging from 24 to 48 h. Thereafter, they were stored in a freezer (−20 °C) until use. GFFs were baked at 500 °C in a muffle furnace for 24 h, placed in pre-cleaned petri dishes and stored in a freezer at −20 °C.

Prior to exposure, all sampling media were defrosted. Afterwards, the adsorber cartridges and PUF discs (including field blanks) were spiked with PRCs. Field blanks were handled and stored like the samples.

All air samples were defrosted prior to extraction, spiked with internal standards and successively extracted by two azeotropic solvent mixtures, namely acetone/hexane (60/40 *v*/*v*) and acetone/methanol (90/10 v/*v*). The extracts were unified and aliquoted. One aliquot was concentrated and solvent exchanged to hexane for GC-MS/MS analysis. The other aliquot was used for additional investigations (Theobald et al. 2013). More experimental details are presented in the supporting material (SM), S1.2 and S1.3, as well as in Mai (2012) and Theobald et al. (2013).

### Water sampling and sample preparation

One hundred litres of unfiltered water was taken by a glass bowl at fixed stations from 5 m depth.

Water samples were extracted with 1 L of pentane immediately after sampling; the extraction was done directly in the sampling glass bowl. Before extraction, a solution of deuterated internal standards was added to the water sample. The dried extract (Na_2_SO_4_) was concentrated to ca. 0.1 mL and pre-cleaned on a small SiO_2_ column. The extract (CH_2_Cl_2_/MeOH 20/80) was concentrated and analysed by GC-MS/MS as described for the air samples (SM, S3). The method had been described in more details earlier (Theobald et al. 1995, 2013).

### Quality assurance and quality control

The reproducibility of air sampling data was controlled by simultaneous side-by-side exposures of two PUF disc passive air samplers and two high-volume active air samplers, respectively, at Hamburg-Sülldorf. The calculated maximum error of air sampling data varied between 9 and 27 % depending on the sampling medium, analyte and the type of instrumental analysis. In addition, desorption experiments were carried out by spiking PRCs to the adsorber cartridge prior to sampling. Sampling errors caused by desorption were assessed to be less than 15 % for the average sample volume of 260 m^3^. The stability of the exposed air sampling material during storage in the freezer was controlled and verified by the recoveries of PRCs in field blanks, which were stored for identical time periods as the air samples. Quality assurance of sample preparation was performed by field blanks, laboratory blanks and spike experiments for each air sample preparation sequence. Field blanks were generated at least in triplicate and were used as control for possible contamination sources of air samples during handling in the field, transport, and handling and analysis in the laboratory. Hence, field blanks provided the data (SM, S1.4) for the blank correction of air sample concentrations as well as for the calculation of the limits of quantification/detection (LOQs/LODs). In addition, laboratory blanks were obtained for different critical stages in air sample preparation, namely extraction, evaporation and cleanup. Significant concentrations of the target compounds in laboratory blanks were not observed. Spike control samples were generated for the whole sample preparation procedure (SM, S1.5) as well as for different stages of sample preparation in accordance with the blank data. LOQs were derived from the signal to noise ratio and the field blanks of each target analyte (SM, S1.6).The analytical determinations of the water samples were done within the regular monitoring programme of the Federal Maritime and Hydrographic Agency (BSH), Hamburg, Germany (BLMP 2005; BSH 2013). The BSH and the procedures are accredited by EN/ISO 17025. External quality assurance was proven by regular participation in laboratory performance studies of Quality Assurance of Information for Marine Environmental Monitoring in Europe (QUASIMEME). Generally, more than 90 % of the data had a *Z*-score of <2 and were thus considered acceptable. Specific validation and quality assurance parameters are presented in the SM, sections S1.3–S1.6. Sampling was field blank controlled (SM, section S1.4). Recoveries of spike control samples were mostly between 80 and 110 % (SM, section S1.5). Reproducibilities for the whole method based on replicates were between 5 and 20 %.

### Air mass history

Recent air mass history was investigated by the calculation of backward trajectories, 24 h in time and 7 days in time, as well as arrival heights 200, 500 and 1000 m. The Hybrid Single-Particle Lagrangian Integrated Trajectory (HYSPLIT) model was used (version 4.9, NCEP Global Data Assimilation System meteorological data; Draxler and Rolph 2003). The trajectories do not reflect dispersion and entrainment of air parcels along transport but merely the central path, being more indicative the closer in time and the more stable the flow.

### Fugacities

The direction of the net flux of the diffusive gas exchange of a given compound was derived from the ratio of the fugacities in the water and air phases, fugacity ratio (FR) = *f*_w_/*f*_a_ (Paterson et al. 1991; Bidleman and Connell 1995). The method is explained in the SM, S1.7.

## Results and discussion

### Chlorinated benzenes

**Fig. 1:**
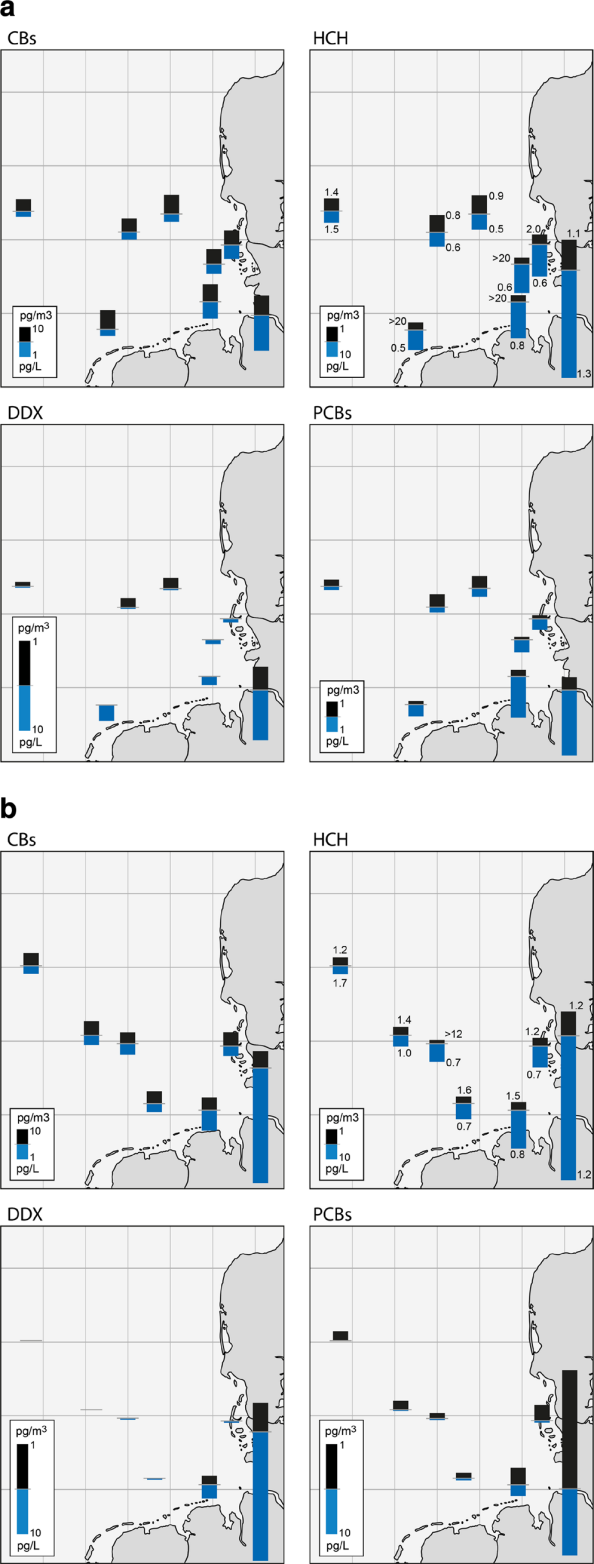
Air and seawater concentrations determined in the German Bight in **a** May/June 2009 and **b** May 2010. Values in the HCH concentration distribution denote the isomer ratio α-HCH/γ-HCH in air and surface water, respectively

**Fig. 2:**
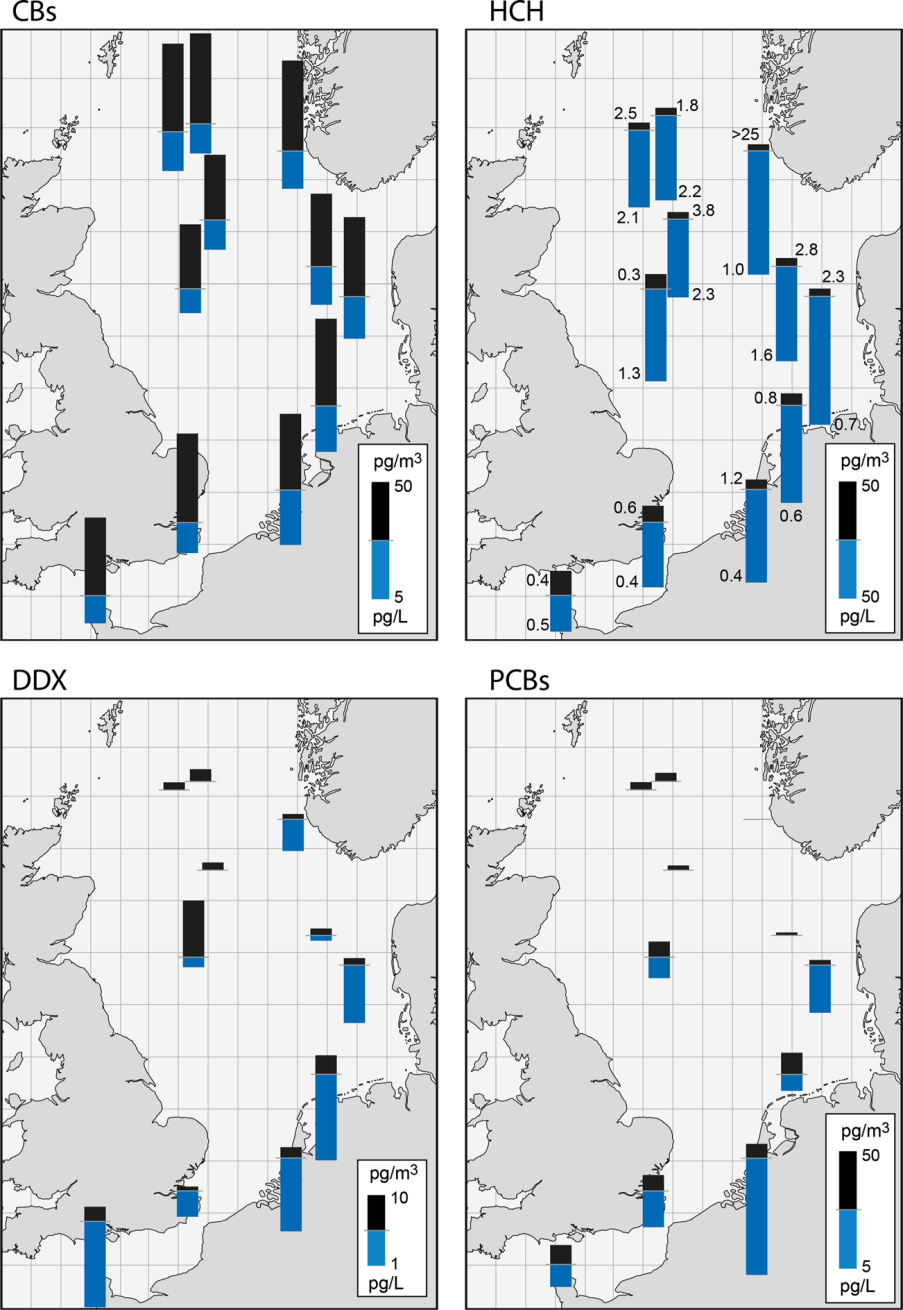
Same as Fig. Fig. 1 but in the wider North Sea in August/September 2009

Hexachlorobenzene (HCB) was identified in all air samples, by average 58 pg/m^3^. The particulate mass fraction of this compound was *θ* = 0.00 (sample volume weighted average). HCB was found to be homogeneously distributed (Figs. Fig. 1 and Fig. 2; Table S6) without significant dependence on the sampling sites or the air mass backward trajectories (SM, Figs. S1–S2). Seasonal fluctuations in its atmospheric concentrations were not observed; monthly passive air sampling data at Hamburg-Sülldorf were 9.2 ± 1.2 ng/sample (mean ± standard deviation, data not shown). A similar distribution pattern was documented in surface seawater (Figs. Fig. 1 and Fig. 2; Table S7). HCB was found throughout all water sampling sites in the German Bight, as well as the wider North Sea, with a the concentration of 2–5 pg/L beyond river plumes. In addition, the concentration gradient in the German Bight emerging from the riverine input of the Elbe was found smaller than for other pollutants. Maximum concentrations of 6–13 pg/L were quantified in the river plume of the Elbe. The small concentration gradients of HCB within the German Bight (and its homogeneous distribution in the surface water of the wider North Sea) pointed to a predominant atmospheric deposition of HCB and to the riverine input having little importance.

The HCB concentrations found in air are both comparable to other sites in the marine environments of Europe (Table 1) as well as to the European median background, which was determined to be 45 pg/m^3^ in the summer of 2006 (Halse et al. 2012).

**Table 1:** OCP and PCB in air in the North Sea region, (a) cruises, (b) coastal and near coastal sites, and arithmetic means (min-max) (pg m^−3^ total concentration, i.e. sum of gas and particulate phases)

	Period (number of samples)	HCB	α-HCH + γ-HCH	dieldrin	*o*,*p′*-DDT + *p*,*p′*-DDT + *p*,*p′*-DDE + *p*,*p′*-DDD	PCB28 + PCB52 + PCB138 + PCB153	
a)							
German Bight (2 cruises, 2 sites)	Aug 1999 (1)	106					Lakaschus et al. (2002)
	Mar 2001 (1)	23					
British Channel (1 cruise, 2 sites)	Oct 2005 (2)					89 (70–107)	Gioia et al. (2008b)
German Bight (2 cruises, 11 sites)^b^	May–June 2009 (9)	55 (44–74)	9.2 (4.6–20.3)	2.0 (<0.9–2.8)	1.2 (<0.8–5.6)	3.1 (2.6–9.1)	This work
	May 2010 (7)	71 (61–88)	6.3 (3.1–16.2)	0.6 (<0.5–1.8)	0.8 (<1.0–6.8)	1.8 (2.1–26.5)	
Central North Sea (1 cruise, 13 sites)^b^	Aug–Sep. 2009 (13)	57.7 (48.5–70.0)	11.2 (<5.8–20.8)	4.0 (1.5–13.0)	5.1 (2.0–16.8)	10.2 (<3.9–19.4)	
German Bight (3 cruises, 7 sites)	Mar–Jul 2010 (19)	56 (7–180)	17 (1–75)				Zhong et al. (2012)
b)							
Westerheversand, Germany (54° 22′ N/8° 38′ E)	Feb–Mar 1996 (5)	41 (34–48)	74 (21–100)			89 (42–172)	Gerwig (2000)
Hamburg (urban, 53° 31′ N/10° 07′ E)	Feb–Apr 1998 (5)	82 (72–123)	408 (51–1429)			147 (50–339)	
Strath Vaich (background, Scotland, 57° 44′ N/4° 46′ E)	Jul–Oct 2006 (1)^a^	42	34		1.7	2.9	Halse et al. (2011)
Auchencorth Moss (rural, Scotland, 55° 50′ N/3° 20′ E)	Jul–Oct 2006 (1)^a^	40	115		4.1	9.1	
High Muffles (England, 54° 20′ N/0° 48′ E)	Jul–Oct 2006 (1)^a^	51	94		10.8	9.3	
Birkenes (Norway, 58° 23′ N/8° 15′ E)	Jul–Oct 2006 (1)^a^	46	16		2.4	6.0	
Tange (rural, Denmark, 56° 21′ N/9° 36′ E)	Jul–Oct 2006 (1)^a^	58	22		6.7	14.7	
Westerland/Sylt (German Bight, 54° 46′ N/8° 19′ E)	Jul–Oct 2006 (1)^a^	46	85		27	25.5	
Kollumerwaard (Netherlands, 53° 20′ N/6° 17′ E)	Jul–Oct 2006 (1)^a^	59	90		36	31.7	
Koksijde (Belgium, 51° 07′ N/2° 29′ E)	Jul–Oct 2006 (1)^a^	52	124		27	38.2	
Lista (Norway, 58° 06′ N/6° 34′ E)	1999 (52)	83 (49–138)	59 (11–196)				EMEP (2012)
	2000 (52)	54 (42–76)	43 (13–126)				
Birkenes (58° 23′ N/8° 15′ E)	2009 (23)	42 (26–56)	10.3 (4.1–19)		2.1 (0.9–4.0)		
	2010 (53)	51 (27–80)	9.8 (3.4–30)		2.4 (0.7–7.0)	1.8 (0.2–7.8)	
Hamburg (residential, 53° 35′ N/9° 47′ E)	Oct 2009–Dec 2010 (15)^c^	34 (14–45)	30 (11–76)	2.5 (<1–7)	9 (3–18)	17 (5–44)	This work
Tinnum/Sylt (German Bight, 54° 54′ N/8° 20′ E)	Oct 2009–Dec 2010 (15)^c^	10 (7–15)	4 (2–7)	1.1 (<0.5–2)	2 (1–6)	3 (2–5)	

For means, values <LOQ were replaced by LOQ/2

^a^Passive sampling, equivalent air volume inferred from performance reference compounds

^b^Volume weighted mean

^c^Passive sampling, equivalent air volume inferred from dependence on wind speed (10 and 40 m^3^ d^−1^ at 4 and 7 m s^−1^, respectively; Tuduri et al. 2006)

The calculation of the net-flux direction of diffusive gas exchange indicated a close to phase equilibrium of HCB within the river plume of the Elbe (FR = *f*_w_/*f*_a_ = 1.0–1.6), with a tendency towards net deposition for the western areas of the German Bight (FR = 0.4–0.5) and the wider North Sea (FR = 0.4–0.7). We used the Tinnum/Sylt data set to investigate whether the concentration in air was controlled by local relaxation to the air-water phase equilibrium. In such a case, the temperature dependence of concentration in the gaseous phase (vapour pressure) would follow the Clausius-Clapeyron equation (ln p = −Δ*H*_exp_/RT + *b*; Hoff et al. 1998; detailed in SM, S2.2). This data set is suitable as it covers a wide range of ground temperatures and, according to the prevailing wind direction, the site should monitor air subject to air-sea gas exchange. The observed slope of the linear regression was far from the value of enthalpy of volatilisation from the liquid phase (see SM, S2.2), indicating that concentrations were not controlled by volatilisation from sea surface but instead by long-range transport (Hoff et al. 1998).

In summary, the results of this study confirmed earlier findings that HCB, in the atmospheric environment across Europe, is homogeneously distributed (Jaward et al. 2004; Halse et al. 2011; Table 1) and close to phase equilibrium in the marine environment in areas with continental influence (Lohmann et al. 2009; 2012).

No such conclusion can be drawn for pentachlorobenzene (QCB). Although QCB was found in homogeneous concentrations of 13 pg/m^3^ throughout the marine atmosphere (*θ* = 0.00; Figs. Fig. 1 and Fig. 2; Table S6), concentrations ranging from 1 to 5 pg/L were found in the surface water of the German Bight and the river delta of the Rhine/Maas/Schelde only (Figs. Fig. 1 and Fig. 2; Table S6). A minor riverine input by the Elbe was indicated by the small concentration gradient within the German Bight.

The calculation of the direction of diffusive gas exchange revealed ambiguous results: Net deposition was indicated for the German Bight in 2009 (FR = 0.2–0.5), whereas close to phase equilibrium was suggested in 2010 (FR = 0.8–2.0) throughout all sampling sites. Differences in temperatures between these two episodes (by average ≈1 K colder in 2010) would support change in flux direction. However, air-sea exchange of POPs is controlled by the combination of the parameters temperature, wind speed and concentration in surface water (Stemmler and Lammel 2011). Moreover, it has been observed that the direction of air-sea exchange may fluctuate once the substance concentration has approached (or is close to) phase equilibrium on a range of time scales (Jantunen and Bidleman 1995; Stemmler and Lammel 2009; Berrojalbiz et al. 2014; Mulder et al. 2014; Lammel et al. 2016). This could explain these observations. It has been pointed out (Lammel et al. 2016) that longer observations (and those across seasons) of the flux is needed to assess the state of air-sea exchange of such substances. Atmospheric deposition of QCB to the wider North Sea could not be documented, because of concentrations below LOQ in most surface water samples. However, net deposition of QCB could not be excluded because of its spatially, almost invariant, abundance in North Sea air.

### Hexachlorocyclohexanes

Hexachlorocyclohexanes were found in the gaseous phase of the atmospheric samples only (particulate mass fraction *θ* < 0.02). β-HCH was scarcely detected, then mostly <LOQ, whereas α-HCH and γ-HCH were quantified at most of the sampling sites (Table S6), with α-HCH/γ-HCH = 1.4 ± 0.8 (mean ± standard deviation, data shown in Figs. Fig. 1 and Fig. 2).

**Fig. 3:**
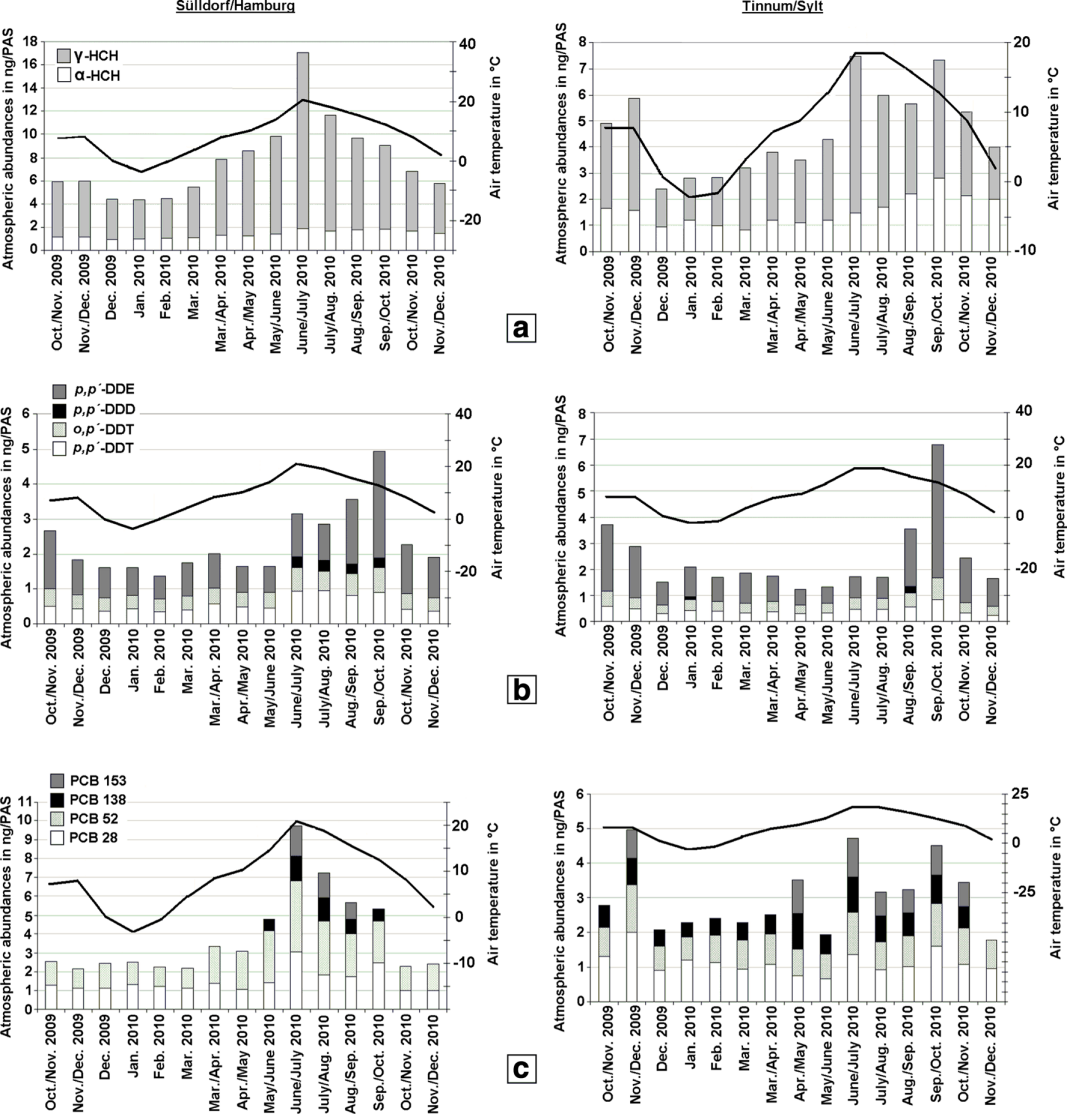
Seasonal variations of α-HCH and γ-HCH (**a**), DDT isomers and metabolites (**b**) and PCBs (**c**) together with ambient air temperature at Sülldorf/Hamburg (*left*) and Tinnum/Sylt (*right*)

α-HCH was homogeneously distributed in the marine atmosphere exhibiting concentrations of 4.90 ± 1.78 pg/m^3^. Its spatial distribution was found to be unaffected by season and the air mass history (Figs. Fig. 1 and Fig. 2; Table S6), including even air mass histories in the North Atlantic (Fig. Fig. 2; Table S6b: PE15, PE16; air mass history of the last 7 days in Fig. S2). This is in agreement with model results which showed that α-HCH is more homogeneously distributed in the seawater of the North Sea. This is in contrast with the γ-HCH distribution (Ilyina et al. 2008). This might be related to the outgassing of α-HCH from the North Atlantic and ice-free regions of the Arctic Ocean reported in previous studies (Bidleman et al. 1995; Bottenheim et al. 2004). Deviations from a fairly constant background level observed over the North Sea were exclusively detected above the river Elbe (Fig. Fig. 1; Table S6: 09AT2, 10AT1), where the concentrations of α-HCH were higher by a factor of 2–3. This could be attributed to the proximity of this sampling site to land, where stronger secondary emission sources (e.g. revolatilisation from contaminated soil) could be expected. By contrast, the concentrations of γ-HCH in the marine atmosphere varied in the range 1–16 pg/m^3^. In addition, a strong dependency of the γ-HCH abundances on the air mass history could be documented (Figs. Fig. 1 and Fig. 2, Table S6): Highest concentrations were quantified in air masses which recently (<24 h back) had passed over England (Fig. Fig. 2; Table S6: PE7, PE10) or France (PE4, PE6). However, a definite source region was not indicated. This was anticipated considering the pronounced multi-hopping potential of this substance (Semeena and Lammel 2005) and recent analysis of its secondary sources in Europe (Dvorská et al. 2009). In general, the γ-HCH concentration increased the longer the air masses had passed over the continent, while in air without significant continental influence, a concentration of <4 pg/m^3^ was determined. This is expected as elevated levels prevail over land of central and western European countries, like Germany and the UK (Table 1). The strong influence of re-volatilisation of γ-HCH from contaminated soils is suggested by the seasonal profile, which shows a strong correlation with ground temperature at Tinnum/Sylt (*r* ^2^ = 0.86) and Hamburg-Sülldorf (*r* ^2^ = 0.91) (Fig. Fig. 3a). Similar observations were made at a central European background station (e.g. Holoubek et al. 2007). In contrast, no significant seasonal variation of α-HCH abundances was observed (maximum amplitudes of 1–2 ng/PAS).

The HCH concentrations found in this study, particularly in the past, are lower than at most background sites in Europe (Table 1). These are clearly below, namely ca. one fourth of the European median background which was 40.1 pg/m^3^ in summer 2006 with α-HCH/γ-HCH ≈ 1.1 (Halse et al. 2012), i.e. lower than observed here over the open North Sea (Fig. Fig. 2).

Corresponding to their ubiquitous presence in the marine atmosphere, α-HCH and γ-HCH were also the main isomers in the surface seawater and could be quantified throughout all water-sampling sites of the German Bight and the wider North Sea. In addition, β-HCH was quantified in all surface water samples (Table S7; sites mapped in Fig. S3b). Compared to earlier measurements in the region (BSH 2009, 2013), the declining trends of α-HCH and γ-HCH with half times of ≈4 years in the period 1989–2005 have been continuing for γ-HCH but somewhat levelled off for α-HCH (similar concentrations as 2003–2005). Regarding the total of HCH isomers in surface water, a decreasing concentration with increasing distance along the sea currents to the river estuaries and the outflow of the Baltic Sea was observed (Fig. S3c). Maximum spatial concentration gradients between the Elbe estuary and the wider North Sea of ≈3000 pg/L (Fig. Fig. 1; Table S6 in combination with Fig. S3a) and ≈100 pg/L (Fig. Fig. 2; Table S7c: sampling sites 909 and 911), respectively, were found. Thus, rivers and the outflow of the Baltic Sea were found to be important sources of HCHs to the North Sea. The main HCH isomers (α-HCH and γ-HCH) displayed inverse spatial trends. Values of α-HCH/γ-HCH were 0.5 for Atlantic water entering the North Sea by the channel (Fig. Fig. 2). In the southern North Sea, the α-HCH/γ-HCH ratio is further decreased to 0.4 inside the river plumes of the Thames and the Rhine (Fig. Fig. 2). However, the ratio gradually increased towards the northern North Sea, achieving maximum values of 2.1–2.3 at the northernmost sites sampled (Fig. Fig. 2). Thus, 4–6 times higher α-HCH than γ-HCH concentrations were quantified at the remote sampling sites in the northern North Sea (Fig. Fig. 2). These observations were supported by the α-HCH/γ-HCH ratios found in the German Bight and immediate vicinity: The ratio increased towards the western sampling sites to a maximum of approximately 1.7, while the ratios in the inner German Bight were 0.4 (Fig. Fig. 1). This is quite remarkable as the ratio at the freshwater border of the river Elbe is ≈2.4 (Table S7), which is caused by a specific historic burden, originating in the middle course of the river (ARGE Elbe 2005). Evidently, the water inflow of the Elbe influences a relatively narrow plume region along the North Frisian coast (eastern boundary of the German Bight) only. The observed elevated concentrations of γ-HCH in the southern North Sea can be explained by several superimposed trends. First, γ-HCH is currently used for medical treatments and has been applied as pesticide (lindane) from the 1980s to the 1990s, whereas α-HCH predominantly originates from the usage of technical HCH in earlier years (Vijgen et al. 2010). Thus, γ-HCH has been discharged in river plumes and estuaries, historically later than α-HCH. In the German Bight, a clear temporal decline was observed for γ-HCH only since 1998, whereas α-HCH showed downward trends since the mid-1980s (BSH 2009). Secondly, α-HCH concentration is higher when it originates from primary emission (55–80 % in technical HCH, α-HCH/γ-HCH = 5–7; IHPA 2006; Vijgen et al. 2010). Although it is similarly resistant to microbial degradation in water (Lammel et al. 2007), it is more resistant to photooxidation (Brubaker and Hites 1998) than γ-HCH. Alongside this, a transformation of γ-HCH to α-HCH was observed (Faller et al. 1991). Consequently, the α-HCH abundances can be expected to exceed those of γ-HCH with increasing distance to riverine input. Moreover, atmospheric deposition might also affect the spatial distribution of α-HCH and γ-HCH in surface seawater, because both were ubiquitously present in the marine atmosphere. A similar spatial trend for the α-HCH/γ-HCH ratio was found earlier in the region (UK and Norwegian sites; Ockenden et al. 1998). The spatial gradients in Europe seem to be somewhat steeper for γ-HCH than for α-HCH (e.g. Halse et al. 2011), possibly influenced by its shorter history of environmental cycling.

The calculation of the net flux of diffusive gas exchange indicated net deposition of γ-HCH into the German Bight and the wider North Sea in spring and summer 2009–10 (FR = 0.1–0.9), whereas close to phase equilibrium could be determined for the Elbe estuary and its river plume (FR = 0.8–1.5). This is in agreement with model results (O’Driscoll et al. 2013). Net atmospheric deposition (FR = 0.2–0.3) of α-HCH in summer was exclusively found for the southern part of the North Sea (adjacent and inside the English Channel). Phase equilibrium (FR = 0.6–1.5) of α-HCH was determined in the German Bight and the wider North Sea in spring and summer. At Tinnum/Sylt (German Bight), neither the α-HCH nor γ-HCH concentrations in air were controlled by local relaxation to (liquid vapour) phase equilibrium (Clausius-Clapeyron equation), even in spring-summer. The observed slope of the linear regression was far from the values of the enthalpy of volatilisation, Δ*H*_vap_ = 67 and 74.7 kJ mol^−1^, respectively for α-HCH and γ-HCH, again indicating that concentrations were not controlled by volatilisation from the sea surface (details in SM, S2.2). Therefore, advection/long-range transport must have been the main source of HCH. Both experimental and modelling studies found that α-HCH and γ-HCH volatilise from the surface seawater of the Elbe estuary in summer (Bethan et al. 2001; Ilyina et al. 2008). Net volatilisation was not found in this study in spring. However, volatilisation could occur in summer (due to higher temperatures) while long-range transport may sustain the concentrations observed (in spring).

In summary, a close to phase equilibrium of α-HCH was observed in the marine environment of the North Sea in spring and summer of 2009 and 2010. This was indicated by the constant atmospheric concentrations unaffected by air mass history, the gradually increasing surface water concentrations towards the northern North Sea and the comparison of air and water fugacities. By contrast, a net dry gaseous deposition was determined for γ-HCH for the same sampling interval. Revolatilisation of γ-HCH from contaminated soil and subsequent transport by advection were observed to be an important source to the marine atmosphere. In addition, riverine input and the Baltic Sea outflow were still major input pathways of HCHs for surface seawater. The local cycling of β-HCH could not be assessed, because its atmospheric and water concentrations are very low (often <LOQ and LOD).

### Cyclodiene pesticides

The concentrations and spatial distribution of dieldrin in the atmosphere of the German Bight and the wider North Sea could be reported in this study, whereas those for aldrin, endrin and isodrin were <LOD throughout (Table S6). Dieldrin was found exclusively in the gas-phase (*θ* = 0.01), at concentrations varying between 1.5 and 3.5 pg/m^3^. Concentrations <LOQ were caused by sample volume limitation. Seasonal variations in atmospheric concentrations were not observed. However, two air samples collected in the wider North Sea (sites PE7 and PE10 in Fig. S2) in August/September 2009 exhibited remarkably increased dieldrin concentrations of 11.4 and 13.0 pg/m^3^, respectively. The air mass history indicated a common source region in central England.

In correlation to its atmospheric abundances, dieldrin was also widespread distributed in the surface seawater of the German Bight and the wider North Sea, whereas endrin and isodrin were only observed in 3 and 1 water sample, respectively, and aldrin was never detected (Table S7). The surface seawater concentrations of dieldrin varied between 3.1 and 19.6 pg/L. Highest concentrations were quantified inside of the river estuaries and river plumes indicating at least a slight riverine input. However, unusually small concentration gradients (in comparison to other pollutants) from the rivers Elbe and Rhine (Fig. S3d) towards the open sea pointed to a pre-contamination of background waters. This could be attributed to atmospheric deposition. Further evidence on atmospheric deposition was given by the comparably high dieldrin concentrations of 9–11 pg/L in the central southwestern part of the North Sea (sampling sites 17, 9, 20). These were obviously unaffected by riverine input considering the sea current profile of the wider North Sea. The remarkably elevated concentrations of dieldrin in the marine boundary layer atmosphere were also observed in this sea region.

The direction of diffusive air-sea gas exchange of dieldrin was net depositional (FR = 0.1–0.2) in the central southwestern part of the North Sea and close to equilibrium adjacent to the exchange regions with the Atlantic Ocean (English Channel/northern North Sea: FR = 0.4) and other parts, consistent with the almost homogenous atmospheric concentrations in spring/summer 2009 and 2010.

### DDT isomers and metabolites

*o*,*p′-*DDT, *p*,*p′-*DDT and the metabolite *p*,*p′-*DDE were quantified mostly in the gas phase of the air over the German Bight and the wider North Sea (*θ* = 0.00, 0.03 and 0.03, respectively; Figs. Fig. 1 and Fig. 2; Table S6), whereas the concentrations of the metabolite *p*,*p′-*DDD were always <LOQ. The metabolite *p*,*p′-*DDE and the parent compound *p*,*p′-*DDT were found to be the main components, ranging 0.7–13 and 0.4–3.6 pg/m^3^, respectively. These values are the first determined in the North Sea and are comparable to others found in the marine environment in the region (Table 1). The DDX concentrations were found to be on average 1.1 and 5.0 pg/m^3^ over the German Bight and the North Sea, respectively. These are similar to the European median background which was 3.8 pg/m^3^ in summer 2006 (Halse et al. 2012) and to the findings in the open Mediterranean (2.6 and 2.7–5.2 pg/m^3^ in the summers 2010 and 2012; Mulder et al. 2015; Lammel et al. 2015).

Whenever observed, the *o*,*p′-*isomer was quantified in almost the same concentration as *p*,*p′-*DDT. Such an isomer ratio is uncommon in Europe: *p*,*p′*-DDT/*o*,*p′*-DDT ≈ 3.6 was found as a long-term mean at a central European background site, Košetice (Holoubek et al. 2007). *p*,*p′*-DDT/*o*,*p′*-DDT ≈ 2 and ≈6 were found in the marine boundary layer of the Mediterranean (Mulder et al. 2015) and in free tropospheric air sampled in the Alps (Lammel et al. 2009), respectively. Such values are closer to the isomer ratio upon primary emission as a pesticide (*p*,*p′*-DDT/*o*,*p′*-DDT ≈ 5), while the ratio in DDT released to the environment as an impurity of another pesticide, dicofol, is often lower. In China, this is as low as *p*,*p′*-DDT/*o*,*p′*-DDT ≈ 0.15 (Qiu et al. 2005). However, dicofol marketed in Europe is reportedly low (12 t in Spain in the year 2000, smaller amounts in Portugal, France and the UK; Denier van der Gon et al. 2007), and the DDT impurity in dicofol marketed in Europe is <0.1 %. (Qiu et al. 2005).

Although detected, the DDT isomers and metabolites could be only sporadically quantified in the atmosphere of the German Bight in spring 2009 and 2010. Their atmospheric concentrations were mostly below the LOQ. By contrast, *p*,*p′-*DDT was quantified in each air sample collected in the wider North Sea in summer 2009, whereas *p*,*p′-*DDE was determined in 12 out of 13 air samples. A significant relation to the air mass history was found for two air samples collected in the North Sea atmosphere in summer 2009: the *o*,*p′–*DDT isomers were slightly elevated and *p*,*p*′-DDE reached 12.5 and 13.1 pg m^−3^ (<LOQ −2.5 pg m^−3^ at other sites in the North Sea; see Table S6b) in air masses which had previously passed over central England (Fig. S2: sites PE7, PE10).

A small seasonal increase in the atmospheric abundances of *p*,*p′-*DDE was monitored from September to October at land based sampling sites. *p*,*p′*-DDD was observed during a few months (Fig. Fig. 3b), but no such seasonality was found for the DDT isomers. Local sources can be excluded as no correlation with local ambient temperature was observed, and this seasonality was more pronounced at the coastal site Tinnum/Sylt than at the residential site Hamburg-Sülldorf. The cause for this *p*,*p′-*DDE seasonality is unknown. Possible influences are long-range transport (very distant sources) and the sensitivity of the PAS sampling efficiency to wind velocity (Melymuk et al. 2011; Tuduri et al. 2006). The latter is unlikely, as no increased wind speed was recorded at the site during September–October.

The concentrations and spatial distribution of *p*,*p′-*DDT (*o*,*p′-*DDT was not targeted in surface seawater analysis) and its metabolites were highly variable within the surface seawater of the German Bight, considering the concentrations observed at individual sampling sites of a single research cruise as well as the fluctuations between the research cruises of two successive years (Figs. Fig. 1 and Fig. 2). On average, the sum of the total concentrations (dissolved and SPM bound) of *p*,*p′-*DDT, *p*,*p′-*DDD and *p*,*p′-*DDE inside the river plume of the Elbe were two times higher in May 2010 than during May/June 2009 (966 vs. 523 pg/L at site STADE and 270 vs. 113 pg/L at site MEDEM, Table S7; see Fig. S3a for site location). The spatial distributions in the inner German Bight observed within 2 years displayed differences depending on the SPM content and the salinity (freshwater fraction) of the water samples At the western border of the German Bight, the DDX concentrations are more homogeneous, but very low (close to or below the LOQs). Therefore, steep concentration gradients within the German Bight were observed, ranging 10–50 pg/L at coastal stations (Table S7: sites EIDER, ELBE1) and 0.3–0.5 pg/L in the western German Bight/Central North Sea (sites ENTE3, ENTE1); the steepness of the gradients is mainly determined by the concentrations at the coastal stations. These variations can be attributed to the strong sorption of DDT isomers and metabolites (logK_OW_ = 6–7) to SPM in the water column. Therefore, the particle loads of rivers and re-suspension processes of contaminated sediments, e.g. during storm events and phytoplankton growth, were expected to be important factors influencing these spatial distributions. A trend of increasing surface water concentrations with increasing SPM content was found, although no significant correlation. This could be explained by the heterogeneous SPM distribution (a strong gradient from coastal and estuarine waters) and differences in the chemical composition and thus adsorption characteristics of the SPM. The spatial distribution of *p*,*p′-*DDT, *p*,*p′-*DDD and *p*,*p′-*DDE indicated a major riverine input into the surface seawater of the German Bight and the wider North Sea. Variations in distribution patterns, additionally pointed to an atmospheric input of *p*,*p′-*DDE. In the Elbe plume (Fig. Fig. 1; Table S6 in combination with Fig. S3a), the concentration of *p*,*p′-*DDD was approximately two times higher than the concentration of *p*,*p′-*DDE. A continuous approximation of the water concentrations of both metabolites, decreasing towards west was observed. This may be explained by air-sea gas exchange or sedimentation with SPM of the two contaminants. In May 2010, at the western sampling sites (Fig. Fig. 1; i.e. sites ENTE3, DTEND and ENTE1 in Table S7), exclusively, *p*,*p′-*DDE was quantified in the surface seawater, whereas *p*,*p′-*DDD concentrations were <LOQ. The same was observed in the wider North Sea. Although *p*,*p′-*DDD revealed the highest surface water concentrations inside the river plumes and estuaries, only *p*,*p′-*DDE was quantified at the northern sampling sites.

The concentrations of DDT and its metabolites in surface seawater of the German Bight were not significantly lower, but similar to those measured in 2005 (BSH 2009).

The direction of air-sea gas exchange of *p*,*p′-*DDT was depositional, as indicated by concentrations in the surface water <LOQ (i.e. FR < 0.2 throughout the North Sea, assuming even total dissolution of the contaminant). *p*,*p′*-DDE, on the other hand, was depositional in the central North Sea but close to phase equilibrium in the German Bight (at location PE1, see Fig. S2, assuming a mostly even association of the contaminant with SPM); *p*,*p′*-DDE had also been found depositional in cold waters of the Atlantic Ocean (Lohmann et al. 2009) and in warm coastal waters (Singapore; He and Balasubramanian 2010). In contrast, *p*,*p′*-DDE was found close to phase equilibrium in the Aegean Sea (Lammel et al. 2015) and net volatilisation in coastal waters of China (East China Sea; Lin et al. 2012). Again, fluctuation of direction of air-sea exchange of *p*,*p′-*DDE as influenced by temperature, wind and concentration in surface water can be expected.

### PCBs

Four PCB congeners, PCB28, PCB52, PCB138 and PCB153, were targeted in this study and could be predominantly quantified in the gaseous phase (*θ* = 0.06, 0.00, 0.04 and 0.26, respectively; Figs. Fig. 1 and Fig. 2; Table S6). PCB28 was the most frequently detected and concentrations reached the highest atmospheric bulk concentrations of up to 9.1 pg/m^3^. In addition, PCB52 was quantified in most of the air samples and reached in bulk concentrations up to 6.9 pg/m^3^. By contrast, PCB138 and PCB153, low volatility congeners (vapour pressures of 0.15 and 0.14 mPa at 25 °C, respectively; Paasivirta et al. 1999) were observed in significantly lower abundances, between <LOQ and 2.6 pg/m^3^ and <LOQ and 8.2 pg/m^3^, respectively. These values are for the first determined in the North Sea and are much lower than what was observed in the English Channel in 2005 and similar to or lower than found at coastal or near-coastal sites in Europe (Table 1).

The atmospheric PCB concentrations’ variation (Figs. Fig. 1 and Fig. 2) was obviously influenced by continental sources. Lower concentrations were observed with increasing distance to coasts and only PCB28 and PCB52 could be quantified in air masses coming from open waters. The variation of the PAS results for PCB at the residential site, Hamburg-Sülldorf (Fig. Fig. 3), was correlated (*R* ^2^ = 0.85, *p* < 0.01) with air temperature. During the months of higher mean air temperatures (12–21 °C), the atmospheric abundances of PCB28 and PCB52 were significantly increased and even the less volatile congeners PCB138 and PCB153 were quantifiable. At lower temperatures, exclusively, the more volatile congeners PCB28 and PCB52 were quantified in almost constant atmospheric concentrations. The seasonality observed can be explained by the main source, revolatilisation from ground, being enhanced by ground temperature. The same was reported elsewhere in Europe (Halsall et al. 1999; Holoubek et al. 2007; Cabrerizo et al. 2011). By contrast, no significant variation in atmospheric PCB abundances with season could be observed at the coastal sampling site Tinnum/Sylt (Fig. Fig. 3c). The fraction of the higher chlorinated congeners, PCB138 and PCB153, in the mixture was apparently higher at Tinnum/Sylt (0.37 ± 0.08) than at Hamburg-Sülldorf (0.25 ± 0.06). This result is likely biased by differences in LOQ at two sites, which are due to differences in PAS’ sampling efficiency as a consequence of differences in wind speed: Based on the results of wind tunnel experiments (Tuduri et al. 2006), the difference in wind speeds between the two sites (6–9 m/s at Tinnum/Sylt and 3–5 m/s at Hamburg-Sülldorf) corresponds to sampling efficiencies differing by a factor of 4. Limited to those months with concentrations at both sites >LOQ no difference in the mixture is found: For these months, May–October, the fraction of the higher chlorinated congeners was 0.38 ± 0.04 at Tinnum/Sylt and 0.40 ± 0.08 at Hamburg-Sülldorf. The difference between PCB concentrations observed at the various campaigns (Figs. Fig. 1 and Fig. 2; Table S6) is dominated by the seasonality of PCB in air as reflected in the time series measured at Hamburg (Fig. Fig. 3c). Similar observations had been made for a central European background site (Holoubek et al. 2007). PCB levels over the open sea had been reported earlier to be dominated by advection of continental air (e.g. Schreitmüller et al. 1994; Gioia et al. 2008a, b).

The PCB concentrations (total of dissolved and SPM bound) in surface seawater displayed the typical spatial distribution emerging from riverine input and subsequent transport along the prevailing sea currents (Fig. S3c). Highest PCB concentrations (sum of the four congeners) were determined close to the river estuaries of the Elbe (10–26 pg/L; Fig. Fig. 1), the Thames (5 pg/L), the Rhine/Maas/Schelde (13–14 pg/L) and the region of exchange with the Baltic Sea (1–4 pg/L) (Fig. Fig. 2), reflecting the predominant riverine input of PCBs into the surface seawater. The PCBs were observed throughout all water sampling sites in the German Bight, but decreased below LOQ towards the northern North Sea. The spatial distribution of PCBs in the surface seawater was influenced by the SPM concentration in the water column. Highest relative amounts of the higher molecular weight PCBs, (PCB138 + PCB153)/Σ_4_PCB, were found in areas with high SPM abundances (e.g. the outflow of the river Elbe). Accordingly, the high molecular weight congeners’ spatial concentration gradients were steeper than those of the lower molecular weight PCBs. For example in May 2009, PCB138 and PCB153 concentrations ranged from ≈11 pg/L at the coastal site EIDER (see Fig. S3a) to <1 pg/L at the open sea sites ENTE3 and ENTE1; but for the same stations, the PCB28 concentrations decreased from 2.4 to 0.2 pg/L, respectively. The atmospheric concentrations of PCB28 and PCB52 indicate that their levels in surface seawater of the open North Sea were strongly influenced by atmospheric deposition. The mean concentration found over the North Sea, 10.2 pg/m^3^, and the ratio (PCB138 + PCB153)/Σ_4_PCB = 0.28 were similar to the European median background of the congeners addressed here which were 11.2 pg/m^3^ and 0.22 in summer 2006, respectively (Halse et al. 2012).

We refrained from deriving the direction of air-sea diffusive gas exchange from fugacities, because it would have been biased by the large non-dissolved (particle bound) PCB mass fraction in seawater (Ilyina et al. 2008; O’Driscoll et al. 2013). No such observations exist for the North Sea. Downward (depositional) or close to equilibrium (within considerable uncertainty) fluxes had been found in the Baltic Sea in 1999 (Bruhn et al. 2003) and in the North Atlantic Ocean in 2005 (Gioia et al. 2008b). For the North Sea, net volatilisation of PCB153 was predicted throughout most of 1996–2005 based on modelling (Ilyina et al. 2008; O’Driscoll et al. 2013).

To sum up, the results of this study showed that riverine input is a major source of PCBs in the German Bight and the wider North Sea. Atmospheric deposition of the lower molecular weight PCBs (PCB28 and PCB52) was indicated as a major source of surface seawater pollution in the wider North Sea.

## Conclusions

We studied organic pollutants in the North Sea atmospheric environment which are mostly fed by primary and secondary emissions (that have been declining in Europe, and worldwide, since a number of years). Very few such measurements (to compare with) have been reported in the past. This is true even when including coastal and near coastal sites (Table 1).

The long-term trends of atmospheric concentrations of the pollutants α-HCH and DDT (and its metabolites) in the North Sea environment are indicated to level off rather than continue to decline. Under varying or eventually decreasing atmospheric concentrations, the direction of diffusive air-sea mass exchange may reverse and seasonal or historic pollution of persistent substances in seawater may be partly returned to the atmosphere, like observed for a currently used pesticide, trifluralin in the North Sea (Theobald et al. 2013), as well as HCH in Arctic and Antarctic seas (Bidleman et al. 1995; Galbán-Malagón et al. 2013) and PCBs in the Mediterranean (Lammel et al. 2015).

More measurements are needed to establish temporal and even safe spatial trends. Air monitoring, at least at coastal stations, should be complemented by seawater monitoring in order to control atmospheric input of contaminants to the sea and the marine ecosystem.

## Electronic supplementary material

ESM 1 (PDF 1019 kb)
