# Supplementary material for: Persistent organochlorine pesticides and polychlorinated biphenyls in air of the North Sea region and air-sea exchange
Source: Environ Sci Pollut Res Int. 2016 Sep 12;23(23):23648–61. doi: 10.1007/s11356-016-7530-3 (PMC5110590; doi:10.1007/s11356-016-7530-3)
Supplement: Supplementary file 1 — (PDF 1019 kb) [file 11356_2016_7530_MOESM1_ESM.pdf]

Supporting Material

Persistent organochlorine pesticides and polychlorinated biphenyls in the North Sea region and air-sea exchange

Carolin Mai [a], Norbert Theobald [a], Gerhard Lammel [b,c*], Heinrich Hühnerfuss [d]

[a]Federal Maritime and Hydrographic Agency (BSH), Hamburg, Germany

[b]Max Planck Institute for Chemistry, Multiphase Chemistry Department, Mainz, Germany

[c]Masaryk University, Research Centre for Toxic Compounds in the Environment, Brno, Czech Republic

[d]University of Hamburg, Department of Chemistry, Hamburg, Germany

S1 Methodology

S1.1 Preparation for the sample collection

PUF plugs, PUF disks and XAD-2 were rinsed with tap water and underwent Soxhlet-extraction with acetone, hexane and methanol for 12 h, respectively. The pre-cleaned adsorber materials were dried in a vacuum dessicator for periods ranging from 24 h to 48 h. Thereafter, the PUF plugs and PUF disks were wrapped twofold in aluminium foil, sealed in zip-lock plastic bags and stored in a freezer (-20 °C) until use. The XAD-2 resin was transferred to a pre-cleaned glass jar with screw cap and was kept frozen (-20 °C). Adsorber cartridges were assembled under the clean-bench. PUF/XAD-2/PUF adsorber cartridges were equipped with pre-cleaned gauze insertions. A smaller PUF plug (250 mm x 550 mm) was placed above the insertion to prevent clogging of the gauze pores by the XAD-2 resin. Thereafter, 10 g of XAD-2 and a second PUF plug (500 mm x 550 mm) were placed inside the cartridge. The assembled adsorber cartridges were wrapped in aluminium foil, sealed in plastic bags and stored in the freezer (-20°C) until deployment. Glass fibre filters were baked at 500°C in a muffle furnace for 24 h. Subsequently GFFs were separately placed in pre-cleaned petri dishes, which were sealed with parafilm®, wrapped in aluminium foil and stored in a freezer at -20°C.

Prior to exposure the GFFs, adsorber cartridges, PUF disks and their respective field blanks were defrosted. Thereafter, the adsorber cartridges, PUF disks and their field blanks were spiked with PRCs, respectively:, 19.1 ng γ-HCH-$^{13}C_6D_6$, 9.8 ng PCB 30, 7.5 ng PCB 104, 12.7 ng PCB 145, 10.3 ng PCB 204 (as well as 10.0 ng simazine-D_{10}, 10.0 ng prometryn-D_6 and 40.8 ng phenanthrene-D_{10}, 39.0 ng fluoranthene-D_{10}, 41.3 ng benzo[*a*]anthracene-D_{12} which are analysed by HPLC-MS/MS and GC-MS and which are not part of this article). GFF and adsorber cartridge were inserted in their respective solvent-rinsed sampler holders prior to their installation into the HVS. Field blanks were packed and stored in the freezer until extraction in parallel to the samples.

S1.2 Air sample preparation

All air samples were defrosted prior to extraction and spiked with internal standards i.e., ε-HCH, HCB-$^{13}C_6$, PCB52-$^{13}C_{12}$, PCB153-$^{13}C_{12}$, PCB185, p,p´-DDT-D_8 for the GC-MS/MS analysis. The air samples were successively extracted by two azeotropic solvent mixtures, namely acetone/hexane (60/40; v/v) and acetone/methanol (90/10; v/v). PUF disks and adsorber cartridges were Soxhlet extracted (with 350 mL of each solvent mixture for 3 h, respectively), whereas GFFs were eluted by diffusion (with 100 mL of each solvent mixture for 2 h, respectively). Thereafter, the successive azeotropic extracts were unified to a single homogenous solution. The target compounds were quantified using GC-MS/MS. For this purpose, the acetone/hexane/methanol-extract was divided into two aliquot parts. One aliquot was used for GC-MS (not part of this article) and GC-MS/MS. The second aliquot was used for HPLC-MS/MS analysis (not subject of this article). The aliquots were evaporated in a parallel-evaporator (Büchi Labortechnik, Essen, Germany). Acetone was the remaining solvent after evaporation allowing a simple solvent exchange to hexane for GC-MS/MS (GC-MS) analysis and methanol for HPLC-MS/MS analysis by replenishing the samples (1.0 – 1.5 mL) with 40 mL of hexane or methanol. After a second evaporation step, the aliquots were centrifuged for 10 minutes at 3000 rpm. The precipitated pellet was discarded, whereas the supernatant underwent a final clean-up in order to minimize matrix effects and to maintain instrument performances. Silica gel was used as clean-up material for the hexane-aliquot analysed by GC-MS/MS. Precipitation of PUF in the methanol aliquot, which could occur after storage in the freezer, was removed using syringe filters prior to the analysis.

S1.3 Instrumental analysis- Acquisition and quantification methods

For GC-MS/MS analysis a Varian CP-3800 GC coupled to a Varian 1200 Quadrupole MS/MS (both Varian Associates, Sunnyvale, USA), and a CTC Combi PAL autosampler (CTC Analytics AG, Zwingen, Switzerland) was used. For separation a Varian Factor Four Column was applied (VF-5ms, 30 m length, 0.25 µm film thickness; Varian Associates, Sunnyvale, USA). The GC-MS/MS gradient program started at 60 °C, holding for 0.2 minutes after large volume (6 µL) injection. Then the temperature was increased with a rate of 10 °C/min to a temperature of 100 °C, which was hold for 0.1 minutes. Finally, the temperature was raised with a rate of 7 °C/min to 320 °C, which was hold for 3.5 minutes. The total duration of the gradient program was 39.2 minutes.

Helium was used as carrier gas. Mass analysis was performed by segmented MRM.

Certified calibration standards were adopted from the routine mass spectrometry analyses of the BSH monitoring programme. The calibration ranges of target analytes ranged from 0.5 to 80 ng/mL with average internal standard concentrations of 15 ng/mL, respectively.

Data acquisition and quantification parameters are listed in table S1.

Table S1: GC-MS/MS data acquisition and quantification parameters; Ret. = retention time in min.; Ion. = Ionisation type; EI = electron impact; Q1 = Q1 mass (m/z); Q3 = Q3 mass (m/z); CE = collision energy in V; -Q = -Qualifier

Parameter	Ret.	Ion.	Mass transition		CE	Internal standard
			Q1	Q3		
Target analytes:						
Aldrin	20.7	EI	262.8	192.8	30	PCB 52-$^{13}C_{12}$
α-HCH	16.6	EI	181.0	145.1	15	PCB 52-$^{13}C_{12}$
α-HCH-Q	16.6	EI	219.0	182.6	10	PCB 52-$^{13}C_{12}$
β-HCH	17.4	EI	219.0	182.6	10	ε-HCH
Dieldrin	23.6	EI	277.0	241.4	10	PCB 153-$^{13}C_{12}$
Dieldrin-Q	23.6	EI	262.8	192.8	30	PCB 153-$^{13}C_{12}$
Endrin	24.1	EI	262.8	192.8	30	PCB 185
γ-HCH	17.7	EI	219.0	182.6	10	PCB 52-$^{13}C_{12}$
HCB	16.7	EI	283.7	248.7	25	HCB-$^{13}C_{6}$
Isodrin	21.6	EI	193.1	157.6	20	PCB 52-$^{13}C_{12}$
Isodrin-Q	21.6	EI	262.8	192.8	22	PCB 52-$^{13}C_{12}$
o,p´-DDT	24.6	EI	235.1	165.0	20	p,p´-DDT-D_8
o,p´-DDT-Q1	24.6	EI	199.1	163.1	20	p,p´-DDT-D_8
o,p´-DDT-Q2	24.6	EI	235.0	199.6	10	p,p´-DDT-D_8
(followed)						

PCB 28	19.4	EI	258.0	185.9	15	PCB 52-$^{13}C_{12}$
PCB 52	20.3	EI	292.0	221.8	15	PCB 52-$^{13}C_{12}$
PCB 52-Q	20.3	EI	220.0	149.8	15	PCB 52-$^{13}C_{12}$
PCB 138	25.6	EI	359.8	289.5	15	PCB 185
PCB 153	24.9	EI	359.8	289.5	15	PCB 153-$^{13}C_{12}$
p,p´-DDD	24.6	EI	235.1	165.0	20	PCB 185
p,p´-DDD-Q1	24.6	EI	199.1	163.1	20	PCB 185
p,p´-DDD-Q2	24.6	EI	235.0	199.6	10	PCB 185
p,p´-DDE	23.4	EI	317.8	246.0	22	PCB 185
p,p´-DDT	25.6	EI	235.1	165.0	20	p,p´-DDT-D_8
p,p´-DDT-Q1	25.6	EI	235.0	199.6	10	p,p´-DDT-D_8
QCB	13.7	EI	249.8	214.7	15	HCB-$^{13}C_6$
<u>Performance Reference Compds.:</u>						
γ-HCH-$^{13}C_6D_6$	17.5	EI	227.9	190.9	10	PCB 52-$^{13}C_{12}$
PCB 30	17.5	EI	257.9	185.9	20	PCB 52-$^{13}C_{12}$
PCB 104	20.7	EI	325.9	253.9	25	PCB 52-$^{13}C_{12}$
PCB 145	23.3	EI	359.8	289.8	30	PCB 153-$^{13}C_{12}$
PCB 204	27.0	EI	429.7	357.9	30	PCB 185
<u>Internal standards:</u>						
ε-HCH	18.8	EI	219.0	182.6	10	-
HCB-$^{13}C_6$	16.7	EI	289.8	254.8	15	-
PCB 153-$^{13}C_{12}$	24.9	EI	371.9	301.9	25	-
PCB 185	26.4	EI	393.7	358.6	10	-
PCB 52-$^{13}C_{12}$	20.3	EI	304.0	232.1	25	-
p,p´-DDT-D_8	25.5	EI	243.0	207.6	10	-

S1.4 Field blanks

Field blanks of PUF disks, PUF/XAD-2/PUF adsorber cartridges and GFFs were prepared (see supplement 1.1) at least in triplicate for each air sampling campaign. Field blanks were used as control samples displaying possible contamination sources of air samples during transportation, their insertion in respective sampler holders, sample preparation and quantification. Hence, blank correction of air concentration data was based on field blanks. Field blank results were also used to calculate the limits of quantification and limits of detection of air sample analysis (see S.1.6). Field blank data is listed in Table S2.

Table S2: Field blanks in ng/mL air sample extract; n = number of field blanks; $\bar{x}_{arithm}$ = arithmetic mean (ng/mL); $\bar{x}_{med}$ = median (ng/mL); σ = standard deviation (ng/mL)

Type of field blank	PUF disk				GFF				PUF/XAD-2/PUF adsorber cartridge			
	n	$\bar{x}_{arithm}$	$\bar{x}_{med}$	σ	n	$\bar{x}_{arithm}$	$\bar{x}_{med}$	σ	n	$\bar{x}_{arithm}$	$\bar{x}_{med}$	σ
Chlorinated Benzenes:												
HCB	36	0.24	0.25	0.12	10	0.16	0.15	0.02	10	0.19	0.19	0.03
QCB	36	0.18	0.22	0.08	10	0.00	0.00	0.00	10	0.20	0.20	0.02
Hexachlorocyclohexanes:												
α-HCH	36	0.01	0.00	0.03	10	0.00	0.00	0.00	10	0.04	0.00	0.06
β-HCH	36	0.00	0.00	0.00	10	0.00	0.00	0.00	10	0.00	0.00	0.00
γ-HCH	36	0.08	0.00	0.13	10	0.00	0.00	0.00	10	0.22	0.21	0.02
Cyclodienes:												
Aldrin	36	0.00	0.00	0.00	10	0.00	0.00	0.00	10	0.00	0.00	0.00
Dieldrin	36	0.00	0.00	0.00	10	0.00	0.00	0.00	10	0.00	0.00	0.00
Endrin	36	0.00	0.00	0.00	10	0.00	0.00	0.00	10	0.00	0.00	0.00
Isodrin	36	0.00	0.00	0.00	10	0.00	0.00	0.00	10	0.02	0.00	0.07
DDT isomers and metabolites:												
o,p´-DDT	36	0.00	0.00	0.00	10	0.00	0.00	0.00	10	0.00	0.00	0.00
p,p´-DDT	36	0.00	0.00	0.02	10	0.00	0.00	0.00	10	0.00	0.00	0.00
p,p´-DDD	36	0.00	0.00	0.00	10	0.00	0.00	0.00	10	0.00	0.00	0.00
p,p´-DDE	36	0.09	0.00	0.12	10	0.00	0.00	0.00	10	0.00	0.00	0.00
Polychlorinated biphenyls:												
PCB 28	36	0.29	0.29	0.15	10	0.02	0.00	0.05	10	0.14	0.13	0.05
PCB 52	36	0.05	0.00	0.11	10	0.00	0.00	0.00	10	0.15	0.16	0.05
PCB 138	36	0.12	0.00	0.27	10	0.00	0.00	0.00	10	0.22	0.25	0.17
PCB 153	36	0.20	0.28	0.15	10	0.05	0.00	0.11	10	0.20	0.21	0.04

S1.5 Recovery of target analytes in spike control samples

The recoveries from GFFs, PUF disks and PUF/XAD-2/PUF adsorber cartridges were determined by spiking pre-cleaned sampling material with specified amounts of target analytes. Sampling was not executed. Results are listed in table S3.

Table S3: Recovery of target analytes in spike control samples of (a) air and (b) water samples; n = number of spike controls; $\bar{x}_{arithm}$ = arithmetic mean (%); $\bar{x}_{med}$ = median (%); σ = standard deviation (%)

a.

Type of spike sample	PUF disk				GFF				PUF/XAD-2/PUF adsorber cartridge			
	n	$\bar{x}_{arithm}$	$\bar{x}_{med}$	σ	n	$\bar{x}_{arithm}$	$\bar{x}_{med}$	σ	n	$\bar{x}_{arithm}$	$\bar{x}_{med}$	σ
Chlorinated Benzenes:												
HCB	11	99	99	5	9	102	100	14	9	98	99	2
QCB	11	84	91	14	9	110	102	18	9	80	76	12
Hexachlorocyclohexanes:												
α-HCH	11	71	74	23	9	12	12	9	9	71	80	21
β-HCH	11	116	106	34	9	312	260	144	9	116	111	26
γ-HCH	11	73	77	22	9	16	11	13	9	74	78	21
Cyclodiene:												
Aldrin	11	91	89	7	9	79	74	13	9	89	87	6
Dieldrin	11	86	89	12	9	67	72	17	9	87	86	9
Endrin	11	125	120	29	9	82	80	28	9	134	140	38
Isodrin	11	95	95	6	9	96	92	14	9	93	95	9
DDT isomers and metabolites:												
o,p´-DDT	11	100	99	20	9	165	147	54	9	98	102	13
p,p´-DDT	11	98	96	7	9	115	109	18	9	98	96	7
p,p´-DDD	11	96	98	7	9	100	102	19	9	103	106	9
p,p´-DDE	11	108	105	10	9	118	118	24	9	107	109	8
Polychlorinated biphenyls:												
PCB 28	11	98	99	4	9	98	96	12	9	100	101	5
PCB 52	11	101	100	6	9	102	100	13	9	104	104	5
PCB 138	11	102	99	5	9	102	101	15	9	102	103	5
PCB 153	11	102	101	5	9	104	102	13	9	102	100	4

b.

Type of spike sample	PUF disk n	$\bar{x}_{arithm}$	$\bar{x}_{med}$	σ
Chlorinated Benzenes:				
HCB	4	66		21
QCB	4	49		14
Hexachlorocyclohexanes:				
α-HCH	4	106		7
β-HCH	4	87		12
γ-HCH	4	107		7
Cyclodiene:				
Aldrin	4	93		8
Dieldrin	4	83		25
Endrin	4	99		19
Isodrin	4	103		13
DDT isomers and metabolites:				
o,p´-DDT	1	100		
p,p´-DDT	4	93		23
p,p´-DDD	4	77		10
p,p´-DDE	4	92		10
Polychlorinated biphenyls:				
PCB 28	4	116		19
PCB 52	4	94		1
PCB 138	2	101		1
PCB 153	2	93		1

S1.6 Limits of Quantification

The limits of quantification (LOQs) were derived from the signal (S) to noise (N) ratio for each target analyte ($LOQ_{S/N} = S = 3 \cdot N$). In addition, LOQs were calculated from field blanks, whenever detected ($LOQ_{Blank} = X_{Arithm.} + 3 \cdot \sigma$). The higher LOQ was applied to air sample quantification procedure, as listed in table S4. Because of the variations in air sample volumes, the LOQs were calculated and applied to the concentrations in the air sample extracts. In order to give an overview on the corresponding atmospheric concentrations, LOQs were exemplarily calculated for the mean sample volume of 260 m^3.

Table S4: Limits of quantification (LOQs) of (a) air and (b) water samples

a.

LOQ	ng/mL (air sample extract)			pg/m³ (mean air volume of 260 m³)		
	GFF	PUF disk	PUF/XAD-2/PUF adsorber cartridge	GFF	PUF disk	PUF/XAD-2/PUF adsorber cartridge
Chlorinated Benzenes:						
HCB	0.07	0.12	0.08	0.26	0.46	0.30
QCB	0.10	0.09	0.09	0.38	0.36	0.34
Hexachlorocyclohexanes:						
α-HCH	0.10	0.09	0.08	0.38	0.35	0.31
β-HCH	0.12	0.14	0.12	0.47	0.56	0.47
γ-HCH	0.12	0.15	0.12	0.47	0.59	0.45
Cyclodienes:						
Aldrin	0.14	0.12	0.11	0.56	0.46	0.41
Dieldrin	0.12	0.12	0.11	0.47	0.47	0.43
Endrin	0.17	0.17	0.17	0.64	0.64	0.64
Isodrin	0.16	0.16	0.16	0.62	0.63	0.62
DDT isomers and metabolites:						
o,p´-DDT	0.08	0.09	0.08	0.30	0.34	0.30
p,p´-DDT	0.06	0.07	0.06	0.21	0.27	0.21
p,p´-DDD	0.08	0.08	0.08	0.30	0.31	0.30
p,p´-DDE	0.10	0.11	0.10	0.38	0.44	0.38
Polychlorinated biphenyls:						
PCB 28	0.08	0.14	0.07	0.29	0.54	0.27
PCB 52	0.06	0.11	0.09	0.24	0.42	0.34
PCB 138	0.10	0.22	0.16	0.38	0.84	0.60
PCB 153	0.15	0.14	0.09	0.57	0.54	0.33

b.

Type of spike sample	pg/L (water volume of 100 L) Liquid/liquid extractionn
Chlorinated Benzenes:	
HCB	0.1
QCB	0.1
Hexachlorocyclohexanes:	
α-HCH	0.1
β-HCH	0.1
γ-HCH	0.3
Cyclodiene:	
Aldrin	4.0
Dieldrin	1.0
Endrin	2.0
Isodrin	5.0
DDT isomers and metabolites:	
o,p´-DDT	0.5
p,p´-DDT	0.1
p,p´-DDD	0.1
p,p´-DDE	0.1
Polychlorinated biphenyls:	
PCB 28	0.2
PCB 52	0.4
PCB 138	0.5
PCB 153	0.4

S1.7 Direction of diffusive air-sea exchange

The direction of diffusive air-surface exchange flux of organics can be identified by comparing the fugacities (Paterson et al., 1991; Bidleman and Connell, 1995). The fugacity ratio, FR, is calculated as:

$$FR = f_w/f_a = c_w\, H / (c_a R T_a)$$

with gas-phase concentration c_a (ng/m^3), dissolved aqueous concentration c_w (ng/m^3), universal gas constant R (Pa m^3 mol^{-1} K^{-1}), water temperature and salinity corrected Henry's law constant H (Pa m^3 mol^{-1}), and air temperature T_a (K). Values $0.3 < FR < 3.0$ are conservatively considered to not safely differ from phase equilibrium, as propagating from the uncertainty of H and measured concentrations (e.g., Bruhn et al., 2003; Castro-Jiménez et al.,

2012; Zhong et al., 2012). This conservative uncertainty margin is also adopted here, while FR > 3.0 indicates net volatilisation and FR < 0.3 net deposition.

The Henry´s Law constants, H, for the calculation of the net flux of diffusive gas exchange of target analytes were adopted from literature. Table S5 lists values of H for the respective surface seawater temperatures (5 m) of 10 °C in May/June 2009 and May 2010 as well as of 17 °C in August/September 2009. Because of reduced solubility in seawater as compared to freshwater values of H and, hence, FR might be somewhat underestimated.

Table S5: Henry´s law constants, H, applied for the temperature dependent calculation of the net flux of diffusive gas exchange of target analytes between the surface seawater and the marine atmosphere

Target analyte	H [Pa m³ mol^{-1}]		Reference
	10 °C	17 °C	
HCB	16.00	26.40	Shen and Wania, 2005
QCB	24.67	41.80	Shen and Wania, 2005
α-HCH	0.20	0.37	Xiao et al., 2004
γ-HCH	0.08	0.15	Xiao et al., 2004
Dieldrin	0.4	0.68	Shen and Wania, 2005

S2 Results

S2.1 Concentrations in air and surface water

Figure S1: Air samples collected in the German Bight in May/June 2009 (A) and May 2010 (B) with the respective air mass history (black arrows). Grey lines correspond to the ship positions during sampling.

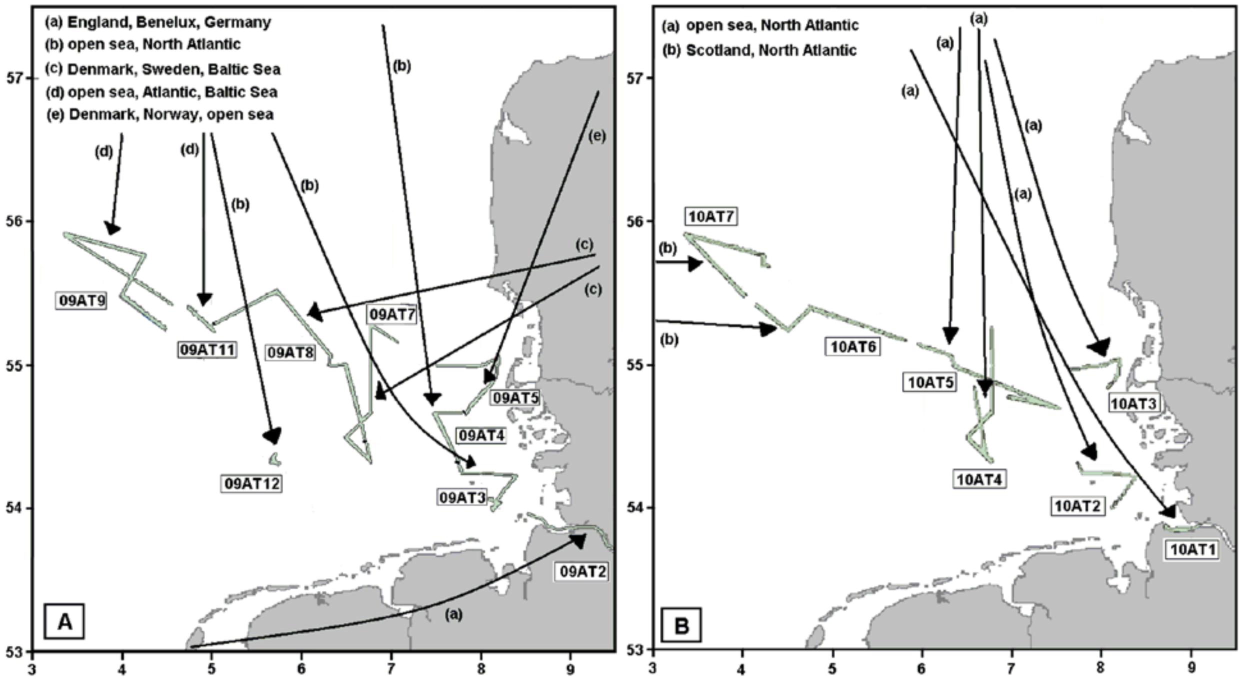

Figure S2: Air samples collected in the wider North Sea in Aug./Sep. 2009 with the respective air mass history (black arrows). Grey lines correspond to the ship positions during sampling.

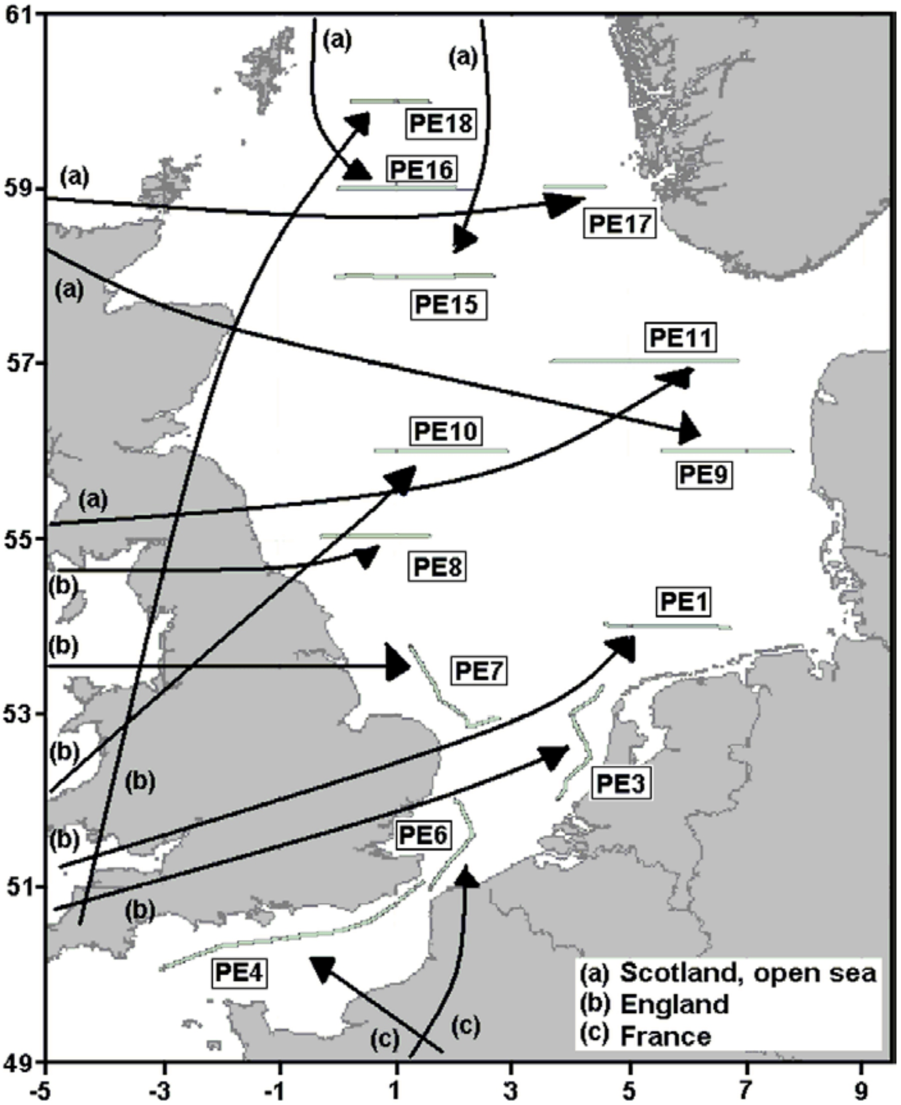

Figure S3: Surface water sampling sites in the German Bight (A) and in the wider North Sea (B); major currents (C); the sampling site STADE is located on the river Elbe at 53.6°N/9.5°W.

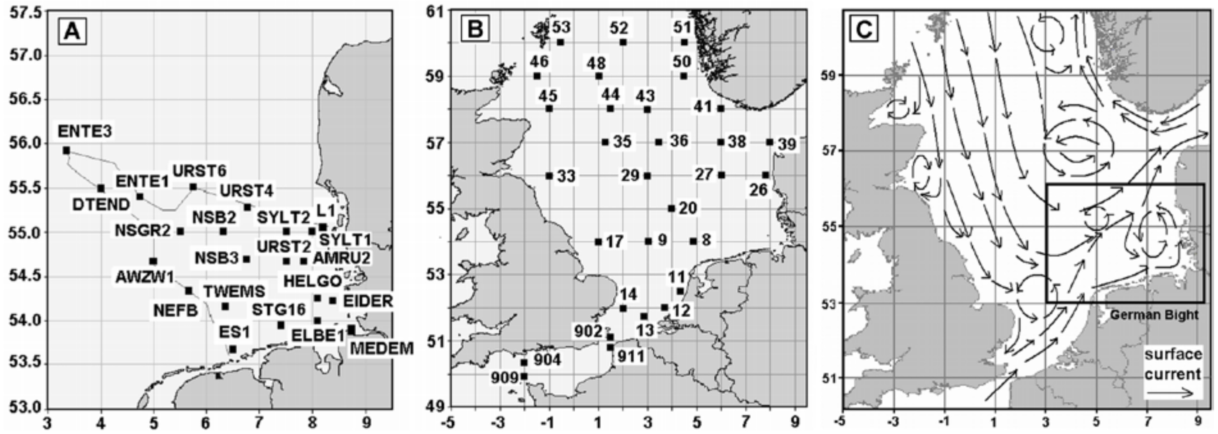

Table S6: Concentrations (pg/m³, sum of gas and particulate phase mass fractions) of OCPs and PCBs in the atmosphere of the German Bight (a) and the wider North Sea (b). For LOQs see Table S4. The sampling sites are mapped in Figures S1 and S2.

	a. German Bight - May/June 2009									German Bight - May 2010						
Air sample	09AT 2	09AT 3	09AT 4	09AT 5	09AT 7	09AT 8	09AT 9	09AT 11	09AT 12	10AT 1	10AT 2	10AT 3	10AT 4	10AT 5	10AT 6	10AT 7
Volume [m³]	144	182	267	241	241	508	414	332	159	159	206	250	306	193	261	254
Chlorinated Benzenes:																
HCB	74.5	62.3	56.4	51.4	72.8	50.7	44.0	48.7	64.0	88.4	65.1	70.5	66.9	61.1	76.4	69.7
QCB	14.7	15.3	12.4	11.6	16.0	9.8	11.7	10.8	14.9	22.2	18.9	20.8	15.1	13.5	18.2	14.7
Hexachlorocyclohexanes[a]:																
α-HCH	10.8	4.3	4.4	4.4	5.9	5.3	4.9	5.1	5.0	8.8	3.3	3.2	3.0	2.7	3.3	3.1
β-HCH	<LOQ	<LOD	<LOD	<LOD	<LOD	<LOD	<LOD	<LOD	<LOD	<LOQ	<LOD	<LOD	<LOD	<LOD	<LOD	<LOD
γ-HCH	9.5	<LOQ	<LOQ	2.2	6.8	6.5	3.4	4.1	<LOD	7.4	2.2	2.7	1.9	<LOQ	2.4	2.6
Cyclodienes[b]:																
Dieldrin	<LOQ	1.8	2.0	2.2	2.3	2.8	1.9	2.3	<LOQ	<LOQ	<LOQ	<LOQ	1.6	<LOQ	1.8	<LOQ
DDT isomers and metabolites[c]:																
o,p´-DDT	<LOQ	<LOQ	<LOQ	<LOQ	<LOQ	0.4	0.5	<LOQ	<LOD	1.3	<LOQ	<LOQ	<LOQ	<LOQ	<LOQ	<LOQ
p,p´-DDT	1.6	<LOQ	<LOQ	<LOQ	0.9	0.5	0.5	<LOQ	<LOQ	1.5	<LOQ	<LOQ	<LOQ	<LOQ	<LOQ	<LOQ
p,p´-DDE	3.5	<LOQ	<LOQ	<LOQ	1.4	1.2	<LOQ	<LOQ	<LOQ	3.8	2.0	<LOQ	<LOQ	<LOQ	<LOQ	<LOQ
Polychlorinated biphenyls:																
PCB 28	5.3	3.0	2.0	2.5	5.9	5.3	3.5	2.2	2.1	8.7	2.3	2.6	1.9	1.2	2.0	2.1
PCB 52	3.3	<LOQ	<LOQ	<LOQ	2.5	2.4	1.3	0.7	<LOQ	10.3	1.5	1.0	<LOQ	<LOQ	<LOQ	<LOQ
PCB 138	<LOD	<LOD	<LOD	<LOD	<LOD	<LOQ	<LOQ	<LOD	<LOD	3.7	<LOQ	<LOQ	<LOQ	<LOQ	<LOQ	<LOQ
PCB 153	<LOQ	<LOQ	<LOQ	<LOQ	<LOQ	1.0	<LOQ	<LOD	<LOQ	3.8	<LOQ	<LOQ	<LOQ	<LOD	<LOQ	<LOD

b. North Sea – August/September 2009

Air sample	PE 1	PE 3	PE 4	PE 6	PE 7	PE 8	PE 9	PE 10	PE 11	PE 15	PE 16	PE 17	PE 18
Volume [m³]	222	297	518	174	251	219	316	284	311	314	230	106	142
Chlorinated Benzenes:													
HCB	64.1	57.3	57.2	67.7	56.0	54.4	58.9	51.6	52.2	48.5	65.2	70.0	68.0
QCB	10.7	8.0	9.2	8.3	8.9	9.5	8.9	9.0	10.3	7.0	10.4	7.8	9.7
Hexachlorocyclohexanes:													
α-HCH	4.6	4.5	5.3	5.2	5.0	4.6	4.7	3.2	5.6	4.9	4.6	5.8	4.5
β-HCH	<LOD	<LOD	0.6	<LOQ	<LOQ	<LOQ	<LOD	<LOQ	<LOD	<LOQ	<LOQ	<LOQ	<LOD
γ-HCH	5.7	3.7	15.2	9.0	15.7	3.2	2.0	9.5	2.0	1.3	1.9	<LOQ	2.5
Cyclodienes[b]:													
Dieldrin	1.8	2.5	3.5	2.3	11.4	3.1	1.9	13.0	2.4	1.5	1.5	2.4	2.6
DDT isomers and metabolites[c]:													
o,p´-DDT	<LOQ	<LOQ	0.7	<LOQ	1.5	<LOQ	<LOD	1.7	<LOQ	<LOD	<LOD	<LOD	<LOQ
p,p´-DDT	2.9	1.5	1.9	1.6	2.0	0.9	0.7	1.9	0.6	0.7	0.8	1.3	1.1
p,p´-DDE	2.5	1.7	1.3	2.5	13.1	2.0	1.3	12.5	1.4	1.4	1.4	<LOQ	2.3
Polychlorinated biphenyls:													
PCB 28	5.8	4.9	6.6	4.9	9.1	2.8	2.5	5.3	2.6	2.2	2.9	<LOQ	2.9
PCB 52	2.5	2.6	4.7	3.3	3.6	2.2	1.3	6.9	<LOQ	2.0	3.7	<LOQ	4.5
PCB 138	1.7	2.5	1.5	2.6	1.8	<LOQ	<LOQ	<LOQ	<LOQ	<LOD	<LOQ	<LOD	<LOD
PCB 153	8.2	2.3	3.5	2.9	4.9	<LOQ	0.7	1.1	<LOQ	<LOQ	0.7	<LOD	<LOQ

[a] δ-HCH was < LOQ in all samples and not displayed

[b] Aldrin, endrin and isodrin were < LOQ in all samples and not displayed

[c] *p,p´*-DDD was < LOQ in all samples and not displayed

Table S7: Concentrations (pg/L) of OCPs and PCBs in surface seawater of the German Bight in (a) May-June 2009, (b) May 2010 and (c) the wider North Sea in August-September 2009; n.a.= not analysed; SPM = suspended particulate matter (mg/L). The sampling sites are mapped in Figure S3.

a1)

Water sampling site	AMRU2	AWZW1	DTEND	EIDER	ELBE1	ENTE1	ENTE3	ES1	HELGO	L1	MEDEM	NSB2	NSB3	NSGR2	STADE
SPM [mg/L]	2.63	0.56	0.65	28.88	11.75	0.09	n.a	n.a	n.a	3.60	20.67	1.13	1.53	0.20	16.55
Chlorinated Benzenes:															
HCB	2.9	2.5	1.8	5.7	4.9	2.1	2.0	4.1	2.9	4.5	13.2	2.2	2.0	2.4	188
QCB	0.8	0.9	0.6	2.4	2.4	0.8	0.9	1.5	0.9	2.4	3.7	0.8	0.8	0.8	30.2
Hexachlorocyclohexanes:															
α-HCH	61.0	35.2	38.9	87.1	45.0	43.7	45.8	41.6	45.5	81.2	204	28.4	25.8	37.2	1290
β-HCH	73.4	8.0	8.4	96.5	23.4	8.3	8.2	23.2	48.8	67.3	299	9.7	10.0	8.7	1330
γ-HCH	85.4	53.8	27.6	112.9	95.9	33.3	24.8	96.9	79.3	99.3	162	56.9	50.8	51.0	510
Cyclodienes[a]:															
Dieldrin	11.9	11.6	7.3	13.7	16.7	8.6	6.2	19.6	9.1	12.8	15.8	9.1	7.0	11.7	11.5
DDT isomers and metabolites[b]:															
p,p´-DDT	1.0	0.3	0.2	1.4	0.7	0.3	0.1	0.7	0.9	0.7	8.8	<LOQ	0.3	0.2	41.8
p,p´-DDD	7.6	0.8	0.4	20.6	5.5	0.4	0.3	2.8	8.5	7.9	87.5	<LOQ	<LOQ	0.6	394
p,p´-DDE	2.4	0.3	0.3	6.9	3.1	0.2	0.1	2.1	2.8	2.7	16.9	0.5	0.6	0.2	88.0
Polychlorinated biphenyls:															
PCB 28	0.9	0.3	0.2	2.6	2.2	0.2	0.2	1.9	1.3	1.1	3.9	0.5	0.5	0.3	13.0
PCB 52	0.6	0.3	<LOQ	1.5	1.3	<LOQ	0.2	1.2	0.9	0.6	3.6	0.5	0.4	<LOQ	17.7
PCB 138	2.6	1.0	2.9	10.8	6.3	0.9	0.9	5.5	4.3	3.3	16.9	1.0	6.3	2.1	48.1
PCB 153	3.1	0.6	1.4	11.5	7.2	<LOQ	<LOQ	5.0	4.8	3.6	19.5	0.7	1.4	<LOQ	50.7

a2)

Water sampling site	SYLT1	SYLT2	URST2	URST4	URST6
SPM [mg/L]	3.80	1.53	1.77	0.92	0.40
Chlorinated Benzenes:					
HCB	4.6	2.9	3.5	3.3	2.5
QCB	3.0	1.0	1.2	1.2	0.8
Hexachlorocyclohexanes:					
α-HCH	79.0	37.2	55.6	35.7	36.9
β-HCH	69.7	28.5	51.5	14.7	8.1
γ-HCH	95.4	60.4	84.7	61.6	43.3
Cyclodienes[a]:					
Dieldrin	14.3	11.7	12.3	12.9	9.4
DDT isomers and metabolites[b]:					
p,p´-DDT	0.8	0.3	0.7	0.3	0.2
p,p´-DDD	7.9	1.8	5.1	1.1	0.5
p,p´-DDE	2.5	0.6	1.7	0.7	0.2
Polychlorinated biphenyls:					
PCB 28	1.2	0.5	0.9	0.6	0.3
PCB 52	0.7	0.3	0.6	0.4	0.2
PCB 138	3.7	1.3	2.0	0.7	<LOQ
PCB 153	4.5	0.8	2.2	0.8	<LOQ

b1)

Water sampling site	AMRU2	AWZW1	DTEND	EIDER	ELBE1	ENTE1	ENTE3	ES1	HELGO	L1	MEDEM	NEFB	NSB2	NSB3	NSGR2
SPM [mg/L]	1.26	0.61	0.30	6.22	3.95	0.06	0.58	6.68	8.15	0.59	25.53	0.70	0.90	1.14	0.12
Chlorinated Benzenes:															
HCB	5.9	3.9	3.9	8.7	12.7	6.5	4.0	6.8	7.4	5.3	55.0	4.4	5.8	4.3	6.3
QCB	1.8	1.1	1.1	3.0	4.9	2.4	1.2	3.0	2.7	1.5	17.9	1.6	1.8	1.3	1.8
Hexachlorocyclohexanes:															
α-HCH	60.1	27.1	34.1	65.5	99.6	41.4	32.1	35.1	59.8	47.9	290	25.6	36.5	36.1	36.2
β-HCH	35.5	6.8	7.0	52.1	90.0	8.9	8.2	25.5	48.8	27.6	271	11.4	12.6	19.9	8.4
γ-HCH	80.7	30.6	32.6	86.7	114.7	39.5	19.0	77.2	80.3	68.4	242	47.2	54.4	56.5	34.8
Cyclodienes[a]:															
Dieldrin	7.5	7.6	6.5	7.8	13.0	11.0	4.3	12.6	9.3	5.9	14.4	12.6	7.9	6.3	11.8
DDT isomers and metabolites[b]:															
p,p´-DDT	1.6	<LOQ	<LOQ	3.4	9.6	<LOQ	<LOQ	1.2	3.6	<LOQ	59.1	0.9	0.5	0.7	<LOQ
p,p´-DDD	3.0	0.3	<LOQ	11.1	29.7	<LOQ	<LOQ	1.6	10.4	2.4	154	1.4	0.5	0.9	0.4
p,p´-DDE	1.9	<LOQ	0.2	5.4	10.5	0.3	<LOQ	2.0	5.5	1.5	56.6	1.1	0.4	0.9	0.2
Polychlorinated biphenyls:															
PCB 28	1.7	0.4	0.4	4.3	6.7	0.6	0.3	3.3	3.1	1.5	20.8	0.7	0.7	1.2	0.7
PCB 52	0.8	n.a.	n.a.	2.2	4.3	n.a.	n.a.	1.8	2.0	0.8	20.4	n.a.	n.a.	0.7	n.a.
PCB 138	2.0	n.a.	n.a.	7.1	10.0	<LOQ	<LOQ	2.7	n.a.	2.1	45.8	1.1	0.6	1.8	0.6
PCB 153	2.5	<LOQ	0.6	8.6	13.3	<LOQ	<LOQ	3.6	9.1	2.4	61.5	1.2	0.6	1.9	0.6

b2)

Water sampling site	STADE	STG16	SYLT1	SYLT2	TWEMS	URST2	URST4	URST6
SPM [mg/L]	3.17	1.28	0.87	0.36	0.83	0.71	0.59	0.31
Chlorinated Benzenes:								
HCB	293	6.0	5.6	4.9	5.9	4.6	4.7	5.2
QCB	30.2	2.7	1.3	1.7	1.4	2.2	0.9	0.9
Hexachlorocyclohexanes:								
α-HCH	1170	47.7	54.0	36.2	52.0	38.2	32.6	29.0
β-HCH	957	32.2	28.2	17.4	32.6	26.1	18.2	6.4
γ-HCH	508	72.7	75.2	55.3	77.3	57.1	47.1	33.0
Cyclodienes[a]:								
Dieldrin	13.2	8.5	7.0	6.1	8.5	6.7	5.9	7.1
DDT isomers and metabolites[b]:								
p,p´-DDT	194	0.8	1.1	0.6	1.0	0.7	0.5	<LOQ
p,p´-DDD	609	3.2	2.3	0.7	3.7	1.3	<LOQ	0.3
p,p´-DDE	163	1.7	1.4	0.7	1.6	1.3	0.5	0.2
Polychlorinated biphenyls:								
PCB 28	36.2	1.7	1.4	0.9	0.9	0.9	0.8	0.5
PCB 52	47.3	1.3	0.7	0.6	0.7	0.5	0.5	n.a.
PCB 138	65.7	1.7	1.8	0.9	0.7	1.4	0.9	<LOQ
PCB 153	82.2	2.2	2.0	1.2	0.9	1.4	1.3	<LOQ

c1)

Water sampling site	8	9	11	12	13	14	17	20	26	27	29	33	35	36	38
SPM [mg/L]	1.02	0.18	0.98	1.41	0.26	5.16	0.24	0.08	0.76	0.19	0.06	0.27	0.17	0.14	0.13
Chlorinated Benzenes:															
HCB	2.8	3.4	3.5	2.9	2.1	2.9	2.6	4.8	3.7	3.4	2.3	2.8	3.7	3.1	3.3
QCB	1.6	<LOQ	3.6	2.0	<LOQ	<LOQ	<LOQ	<LOQ	<LOQ	<LOQ	<LOQ	<LOQ	<LOQ	<LOQ	<LOQ
Hexachlorocyclohexanes:															
α-HCH	27.7	27.2	20.4	16.8	8.3	17.4	26.7	36.1	34.3	37.4	40.2	33.9	41.8	43.6	45.1
β-HCH	11.3	8.6	30.9	9.8	6.3	9.0	9.2	9.4	28.3	8.2	7.7	7.3	8.3	8.2	8.5
γ-HCH	49.9	39.4	62.2	47.6	20.5	43.1	33.0	35.9	64.8	39.0	31.1	23.2	19.1	27.0	27.7
Cyclodienes[a]:															
Dieldrin	15.5	10.9	16.1	12.0	4.3	11.5	9.2	9.8	11.4	9.4	5.8	4.5	5.9	6.3	5.9
DDT isomers and metabolites[b]:															
p,p´-DDT	<LOQ	<LOQ	<LOQ	<LOQ	<LOQ	<LOQ	<LOQ	<LOQ	<LOQ	<LOQ	<LOQ	<LOQ	<LOQ	<LOQ	<LOQ
p,p´-DDD	2.2	0.9	1.8	1.3	<LOQ	2.1	<LOQ	<LOQ	1.2	0.7	<LOQ	<LOQ	<LOQ	<LOQ	<LOQ
p,p´-DDE	1.2	0.6	1.4	1.2	0.6	2.3	0.8	0.5	1.0	0.4	0.3	0.3	0.3	0.3	<LOQ
Polychlorinated biphenyls:															
PCB 28	0.7	0.4	2.5	1.5	0.4	0.7	0.5	0.4	0.6	<LOQ	0.3	0.3	<LOQ	<LOQ	<LOQ
PCB 52	0.4	<LOQ	2.5	1.3	<LOQ	0.6	0.6	<LOQ	<LOQ	<LOQ	<LOQ	<LOQ	<LOQ	<LOQ	<LOQ
PCB 138	0.7	0.6	2.6	<LOQ	0.9	1.4	0.9	0.6	2.4	<LOQ	1.5	<LOQ	<LOQ	<LOQ	<LOQ
PCB 153	<LOQ	<LOQ	6.2	9.9	2.0	2.0	<LOQ	<LOQ	5.3	<LOQ	<LOQ	<LOQ	<LOQ	<LOQ	<LOQ

c2)

Water sampling site	39	41	43	44	45	46	48	50	51	52	53	902	904	909	911
SPM [mg/L]	0.89	0.08	0.08	0.05	0.15	0.05	0.07	0.26	0.23	0.05	0.06	1.46	1.15	0.73	1.21
Chlorinated Benzenes:															
HCB	3.3	2.6	2.0	3.1	3.0	4.2	3.3	3.2	2.5	2.5	2.7	2.3	3.1	1.8	2.1
QCB	<LOQ	<LOQ	<LOQ	<LOQ	<LOQ	<LOQ	<LOQ	<LOQ	<LOQ	<LOQ	<LOQ	<LOQ	<LOQ	<LOQ	<LOQ
Hexachlorocyclohexanes:															
α-HCH	29.0	42.4	32.5	48.1	33.8	42.2	38.1	39.4	35.4	42.0	32.1	10.1	9.8	8.1	7.7
β-HCH	14.7	24.9	8.0	8.3	8.8	9.3	9.4	24.8	25.9	11.8	8.4	6.5	5.2	5.2	5.9
γ-HCH	55.3	39.7	18.1	17.6	12.6	14.3	18.2	41.1	35.2	18.8	12.3	22.2	16.9	13.8	19.9
Cyclodienes[a]:															
Dieldrin	11.0	5.3	3.4	5.2	3.9	4.6	4.8	6.7	5.4	3.6	3.5	4.9	5.2	3.1	3.9
DDT isomers and metabolites[b]:															
p,p´-DDT	<LOQ	<LOQ	<LOQ	<LOQ	<LOQ	<LOQ	<LOQ	<LOQ	<LOQ	<LOQ	<LOQ	<LOQ	<LOQ	0.6	<LOQ
p,p´-DDD	0.7	<LOQ	<LOQ	<LOQ	<LOQ	<LOQ	<LOQ	0.5	<LOQ	<LOQ	<LOQ	<LOQ	<LOQ	<LOQ	<LOQ
p,p´-DDE	0.7	<LOQ	<LOQ	<LOQ	<LOQ	<LOQ	<LOQ	0.4	0.3	<LOQ	<LOQ	0.5	0.3	0.3	0.5
Polychlorinated biphenyls:															
PCB 28	0.3	<LOQ	<LOQ	<LOQ	<LOQ	<LOQ	<LOQ	<LOQ	<LOQ	<LOQ	<LOQ	0.3	0.3	<LOQ	<LOQ
PCB 52	0.3	<LOQ	<LOQ	<LOQ	<LOQ	<LOQ	<LOQ	<LOQ	<LOQ	<LOQ	<LOQ	0.5	<LOQ	<LOQ	0.4
PCB 138	<LOQ	1.8	<LOQ	<LOQ	<LOQ	<LOQ	<LOQ	<LOQ	<LOQ	<LOQ	<LOQ	0.6	0.6	1.2	1.1
PCB 153	<LOQ	2.2	<LOQ	<LOQ	<LOQ	<LOQ	<LOQ	<LOQ	<LOQ	<LOQ	<LOQ	<LOQ	<LOQ	<LOQ	2.0

[a] Aldrin, endrin and isodrin were < LOQ in all samples and not displayed

[b] *o,p´*-DDT was not analyzed

S2.2 Test of significance of air-surface exchange

If the gas-phase concentrations were controlled by local relaxation to (liquid–vapour) phase equilibrium, then the relationship between these and ambient temperature should be described by the Clausius–Clapeyron equation (Hoff et al., 1998):

$$\ln p = -\Delta H_{exp}/RT + b$$

with p (Pa) is the partial vapour pressure in air (derived from concentrations by application of the ideal gas law), and ΔH_{exp} (kJ mol^{-1}) is an enthalpy of the surface-air exchange. The observed ΔH_{exp} should be close to the values of enthalpies of volatilisation from the liquid phase, ΔH_{vap}, if air–water exchange prevailed, and of octanol–air partitioning that represents the soil–air and vegetation–air gas exchanges, ΔH_{oa}.
The respective (so-called Clausius–Clapeyron) plot shows considerable scatter and the slopes of the linear regressions are flat, or even positive (HCB). Note, that ΔH_{exp} (Table S8) is taken from the slope (equal to $-\Delta H_{exp}/R$), which is not affected by unit of the y-axis, which is arbitrary. The unit shown (Fig. S4) results from conversion of trace substance amount measured in air (ng) into pressure (Pa) assuming a unit efficient sampling volume of 4 m^3/day. This efficient sampling volume has often been used for OCPs collected in samplers of the design used and at various kinds of sites (e.g., Gouin et al., 2005), but is sensitive to wind velocity. Seasonal variation of wind velocity might have contributed to the scatter.
The results (Table S8) indicate that liquid–vapour equilibria were not established and gaseous concentrations were controlled by some other process. A large scatter in the data can often be attributed to lack of representativeness of the temperature measurement for the volatilisation process, which is very likely if substance concentration was influenced by long-range transport (other temperature under which volatilisation happened; Hoff et al., 1998).

Fig. S4. Clausius-Clapeyron plots $\ln p = -\Delta H_{exp}/RT + b$ of HCH isomers and HCB at Tinnum/Sylt based on the results of monthly data (passive air sampling, see section 2.1). Conversion into pressure units assuming a unit efficient sampling volume of 4 m^3/day.

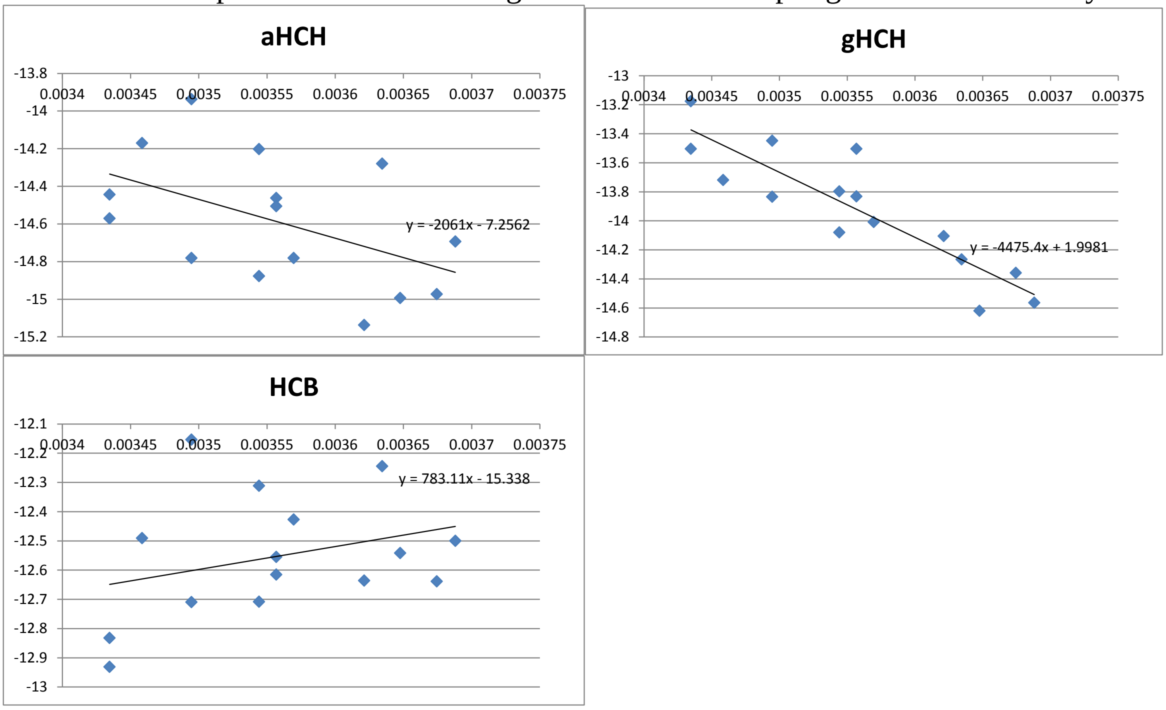

Table S8. Observed values of the enthalpy of the surface-air exchange, ΔH_{exp}, based on Clausius-Clapeyron plots (Fig. S4) of passive air sampling data from Tinnum/Sylt, and literature values of the enthalpy of volatilization from the liquid phase, ΔH_{vap}.

Substance	ΔH_{exp} (kJ mol^{-1})	ΔH_{vap} (kJ mol^{-1})	
α-HCH	+17.1	+67.0	Xiao et al., 2004
γ-HCH	+37.2	+74.7	Xiao et al., 2004
HCB	- 6.5	+67.6	Shen and Wania, 2005

References

Bidleman TF, McConnell LL (1995) A review of field experiments to determine air–water gas-exchange of persistent organic pollutants. Sci. Total Environ. 159, 101-107

Bruhn R, Lakaschus S, McLachlan MS (2003) Air/sea gas exchange of PCBs in the southern Baltic sea. Atmos. Environ. 37, 3445–3454

Castro-Jiménez J, Berrojalbiz N, Wollgast J, Dachs J (2012) Polycyclic aromatic hydrocarbons (PAHs) in the Mediterranean Sea: Atmospheric occurrence, deposition and decoupling with settling fluxes in the water column. Environ. Pollut. 166, 40-47

Gouin T, Harner T, Blanchard P, Mackay D (2005) Passive and active air samplers as complementary methods for investigating persistent organic pollutants in the Great Lakes Basin. Environ. Sci. Technol. 39, 9115-9122

Hoff RM, Brice KA, Halsall CJ (1998) Nonlinearities in the slopes of Clausius-Clapeyron plots for SVOCs. Environ Sci Technol 32:1793-1798

Paterson S, Mackay D, Gladman A (1991) A fugacity model of chemical uptake by plants from soil and air. Chemosphere 23, 539-565

Shen L, Wania F (2005) Compilation, evaluation, and selection of physical-chemical property data for organochlorine pesticides. J. Chem. Eng. Data 50, 742-768

Xiao, H., Li, N.Q., Wania, F., 2004. Compilation, evaluation and selection of physico-chemical property data for α-, β- and γ-hexachlorocyclohexane. J. Chem. Eng. Data 49, 173-185

Zhong G, Xie Z, Möller A, Halsall C, Caba A, Sturm R, Tang J, Zhang G, Ebinghaus R (2012) Currently used pesticides, hexachlorobenzene and hexachlorocyclohexanes in the air and seawater of the German Bight (North Sea). Environ. Chem. 9, 405-414
